# Loss of Fnip1 alters kidney developmental transcriptional program and synergizes with TSC1 loss to promote mTORC1 activation and renal cyst formation

**DOI:** 10.1371/journal.pone.0197973

**Published:** 2018-06-13

**Authors:** Ryan Centini, Mark Tsang, Terri Iwata, Heon Park, Jeffrey Delrow, Daciana Margineantu, Brandon M. Iritani, Haiwei Gu, H. Denny Liggitt, Janella Kang, Lim Kang, David M. Hockenbery, Daniel Raftery, Brian M. Iritani

**Affiliations:** 1 The Department of Comparative Medicine, University of Washington, Seattle, Washington, United States of America; 2 Genomics and Bioinformatics Shared Resources, Fred Hutchinson Cancer Research Center, Seattle, Washington, United States of America; 3 Clinical Division, Fred Hutchinson Cancer Research Center, Seattle, Washington, United States of America; 4 Department of Anesthesiology and Pain Medicine, Mitochondria and Metabolism Center, Northwest Metabolomics Research Center, University of Washington, Seattle, Washington, United States of America; 5 Public Health Sciences Division, Fred Hutchinson Cancer Research Center, Seattle, Washington, United States of America; Universidade de Sao Paulo, BRAZIL

## Abstract

Birt-Hogg-Dube’ Syndrome (BHDS) is a rare genetic disorder in humans characterized by skin hamartomas, lung cysts, pneumothorax, and increased risk of renal tumors. BHDS is caused by mutations in the *BHD* gene, which encodes for Folliculin, a cytoplasmic adapter protein that binds to Folliculin interacting proteins-1 and -2 (Fnip1, Fnip2) as well as the master energy sensor AMP kinase (AMPK). Whereas kidney-specific deletion of the *Bhd* gene in mice is known to result in polycystic kidney disease (PKD) and renal cell carcinoma, the roles of Fnip1 in renal cell development and function are unclear. In this study, we utilized mice with constitutive deletion of the *Fnip1* gene to show that the loss of *Fnip1* is sufficient to result in renal cyst formation, which was characterized by decreased AMPK activation, increased mTOR activation, and metabolic hyperactivation. Using RNAseq, we found that *Fnip1* disruption resulted in many cellular and molecular changes previously implicated in the development of PKD in humans, including alterations in the expression of ion and amino acid transporters, increased cell adhesion, and increased inflammation. Loss of *Fnip1* synergized with *Tsc1* loss to hyperactivate mTOR, increase Erk activation, and greatly accelerate the development of PKD. Our results collectively define roles for Fnip1 in regulating kidney development and function, and provide a model for how loss of Fnip1 contributes to PKD and perhaps renal cell carcinoma.

## Introduction

Birt-Hogg-Dube’ Syndrome (BHDS) is a rare autosomal dominant disorder in which affected individuals are at high risk of developing cutaneous fibrofolliculomas, bilateral pulmonary cysts, pneumothorax, and a variety of renal tumors including chromophobe renal cell carcinoma, clear cell renal carcinoma, oncocytoma, and papillary renal cell carcinoma (see [1, 2] for review). In 2002, BHDS was localized to germline mutations in the *BHD* gene, which encodes a tumor suppressor named Folliculin (FLCN)[3]. Additional protein-protein interaction studies revealed that the carboxy terminus of FLCN binds to two structurally related proteins called Folliculin interacting proteins-1 and -2 (FNIP1 and FNIP2), as well as the master metabolic regulator AMP kinase (AMPK)[4–6]. AMPK is an energy sensing molecule that is activated in response to low energy (low ATP/high AMP) via phosphorylation at threonine 172 by Liver Kinase B1 (LKB1) and by allosteric activation of AMPK upon AMP binding (see [7] for review). In some cell types such as T cells, AMPK is also phosphorylated and activated by Calmodulin-dependent protein Kinase Kinase (CaMKK) in response to calcium signals [8]. Once activated, AMPK stimulates energy production in part by stimulating mitochondrial biogenesis, fatty acid oxidation, glucose uptake, and autophagy, while also inhibiting energy and nutrient consumption by negatively regulating mechanistic target of rapamycin (mTOR). Specifically, AMPK phosphorylates and inhibits the essential mTOR co-activator Raptor[9], and phosphorylates and stimulates TSC2, a negative regulator of mTOR[10]. Whereas the exact roles of Folliculin in modulating these pathways are not clear, Folliculin/Fnip have been implicated in both inhibition and activation of AMPK[11–16], inhibition of mitochondrial biogenesis [17–19], inhibition and activation of autophagy[15, 20, 21], and inhibition and activation of mTOR [18, 22–26].

Constitutive deletion of the *Bhd* gene in mice results in early embryonic death[23]. Hence, the majority of Flcn studies in mice have involved either analyses of *Bhd* heterozygous mice, or conditional deletion of *Bhd* in a tissue specific manner using the Cre-LoxP system. Three independent research groups found that heterozygous *Bhd* (*Bhd*^*+/-*^) mice developed PKD, which progressed to renal cancer with a median tumor-free survival of ~25 months [23, 26, 27]. Similar to BHDS, histological types of tumors from *Bhd*^*+/-*^ mice included oncocytic hybrid, oncocytoma, and clear cell tumors, which were characterized either by increased[23], decreased[26], or variable activation of mTOR depending on the tissue context[27]. Three additional studies whereby the *Bhd* gene was deleted in a kidney-specific manner in either distal[28, 29] or proximal tubules[30] of mice found that the mice initially developed PKD, which progressed to renal failure. These studies showed that loss of the *Bhd* gene resulted in pronounced upregulation of the AKT/mTOR signaling pathways and that treatment with the mTORC complex 1 (mTORC1) inhibitor rapamycin could prevent the development of PKD. In a similar study, *Hasumi* et al conditionally deleted the *Bhd* gene in the proximal tubules using *CDH16*-Cre, and found that the development of PKD correlated with increased *Pgc1alpha* expression and increased mitochondrial metabolism. Disruption of the *Pgc1alpha* partially rescued the PDK phenotype in *Bhd* deficient mice[17]. Collectively, these studies suggest that disruption of Folliculin is sufficient to result in the development of PKD, and can in some cases progress to renal cancer due in part to increased activation of the mTOR pathway and increased mitochondrial biogenesis.

Although the roles and importance of Folliculin in kidney development and function have been extensively investigated, the roles of Fnip1 and Fnip2 in kidney development and function are not as clear. Recently, *Hasumi et al* generated *Fnip1*, *Fnip2* double knockout mice in a kidney specific manner[31]. The authors found no changes in the kidney development or function when either *Fnip1* or *Fnip2* were deleted alone, but when both genes were deleted together, the mice developed fatal PKD by 3 weeks of age. Interestingly, kidney specific deletion of *Flcn* did not augment kidney size or cystic histology of *Fnip1*^*-/-*^*Fnip2*^*-/-*^ double null kidneys, suggesting that Fnip1/2 and Flcn likely act on convergent pathways. The authors also showed heterozygous *Fnip1*^*+/-*^ *Fnip2*^*-/-*^ mice developed hybrid oncocytic tumors at 24 months of age, similar to the tumor type found in BHDS patients.

In this study, we investigated the specific roles of Fnip1 in renal development and function, using *Fnip1* null mice we previously generated using a random ENU mutagenesis strategy in mice [18]. In contrast to a previous report[31], we found that loss of *Fnip1* resulted in slightly enlarged kidney size and significantly increased renal cyst formation, which were characterized by decreased AMPK activation, focal increases in mTORC1 activation, and increased oxidative metabolism of renal epithelial cells. The local cyst environment exhibited increased immune cell infiltration, decreased expression of organic ion channels, and increased expression of cell adhesion molecules. Importantly, co-deletion of the mTORC1 inhibitor *Tsc1* with *Fnip1* resulted in greatly increased mTORC1 activation and accelerated PKD development relative to deletion of either gene alone.

## Materials and methods

### Mice

*Fnip1*^*-/-*^, *Mb1-Cre*, *Tsc1*^*fl/fl*^ mice were developed as previously described[18, 32, 33]. Mice were housed under specific-pathogen-free conditions. *TSC1*^*fl/fl*^*Mb1*Cre, *TSC1*^*fl/fl*^*Mb1CreFnip1*^*+/-*^, *TSC1*^*fl/fl*^*Mb1CreFnip1*^*-/-*^ mice were humanely euthanized upon signs of compromised renal function. Rapamycin and control diets were milled at TestDiet (Richmond, IN) and impregnated with rapamycin (14ppm) in the lab of Dr. Randy Strong at the University of Texas Health Center, Barshop Institute (San Antonio, TX). For the *TSC1*^*fl/fl*^*Mb1*Cre study, mice in the rapamycin treatment group were started on rapamycin chow after weaning and continued until euthanasia. All animal studies were reviewed and approved by the Institutional Animal Care and Use Committee of the University of Washington. Mice were humanely euthanized via CO_2_ overdose according to the AVMA guidelines on euthanasia.

### Immunoblotting

Tissue for immunoblotting and RNA analyses were flash frozen and stored at -80 degrees Celsius until lysis. Frozen kidneys were homogenized in RIPA buffer (Thermo Scientific Rockford, IL) with Halt Protease and Phosphatase Inhibitor^™^ (Thermo Scientific Rockford, IL). Protein concentrations were determined via the Micro BCA^™^ Protein Assay Kit (Thermo Scientific, Rockford, IL). Immunoblotting was performed as previously described[18, 34]. Blots were incubated overnight in primary antibodies obtained from Cell Signaling Technology (Danvers, MA), followed by incubation with horseradish peroxidase-conjugated secondary antibody. Antibodies used were: pERK(D13.14.4E; Rabbit mAb; Cat# 4370), Erk (137F5; Rabbit mAb;Cat#4695), p-AMPK (40H9; Rabbit mAb; Cat#2535), AMPK (Rabbit Ab;Cat#2532, pmTOR (D9C2; Rabbit mAb;Cat#5536), pS6R(D68F8; Rabbit mAb;Cat#5364), p4E-BP (235B4;Rabbit IgG;Cat#2855), 4E-BP(53H11;Rabbit mAb;Cat#9644), pAkt473 (D9E; Rabbit mAb;Cat#4060), pAkt308(D25E6;Rabbit mAb; Cat#13038), Actin (D6A8;Rabbit mAb;Cat#8457).

### Histochemistry and immunohistochemistry

Kidneys were collected and fixed in 10% formalin. All special staining was performed at the University of Washington’s Histology and Imaging Core (HIC). Phospho-S6R (Cell Signaling Technology, Danvers, MA), and F4/80 (Thermo Scientific, Waltham, MA), and anti-CD3epsilon (eBioscience, San Diego CA) antibodies were used. To count cysts, one kidney from 12–16 week-old male mice was fixed in 10% neutral buffered formalin and processed as previously described followed by staining with hematoxylin and eosin [29]. Sagittal sections of equivalent depth and orientation were evaluated by a board-certified veterinary pathologist. Cysts (> 5 x normal tubule diameter) and microcysts (<5X normal tubule diameter) were defined as translucent regions lined by renal tubular epithelial cells with blebs extending into the lumen. Cysts were counted by an individual blinded to the genotypes.

### Quantitative PCR

RNA was purified from frozen kidney tissue with the use of RNeasy Mini Kit (Qiagen Valenica, CA). cDNA was synthesized from RNA via Invitrogen SuperScript II Reverse Transcriptase (Life Technologies Grand Island, NY). Gene-specific oligonucleotide primer sequences are listed in S4 Table. PCR reactions were performed using the SYBR green system as previously described[35]. For real-time quantitative PCR, differences were calculated using the comparative C_T_ method (n = 3/group)[36].

### Seahorse assays

Renal tubular epithelial cells (TECs) were isolated from kidneys collected from 3 *Fnip1*^*-/-*^ and 3 *Fnip1*^*+/-*^ (wildtype) mice. Briefly, kidneys were dissected into 4 mls ice-cold Dulbecco’s phosphate buffered saline (DPBS) (Gibco Grand Island, NY) and minced into pieces of ~ 1 mm^3^. Fragments were then transferred to collagenase solution (1 mg/ml in DPBS; Sigma-Aldrich St. Louis, MO) and digested for 60 minutes at 37 °C. The pellet was washed twice with DPBS before plating in 10 cm dishes to a confluence of 80–90%. Cells were cultured in RPMI media (Gibco, Grand Island, NY), 10% FBS, 100 units/ ml penicillin G, 100 μg/ ml streptomycin, and 20 ng/ ml epidermal growth factor (Sigma-Aldrich St, Louis, MO). Cells were seeded on an XF96 microplate (Seahorse Biosciences) and incubated at 37 degrees Celsius for 6–12 hours prior to analysis. Cells were plated in DMEM +10mM glucose, 2mM glutamine, and 1mM pyruvate. Mitochondrial function was analyzed in accordance with the XF Cell Mito Stress Test Kit (Seahorse Biosciences) and basal respiratory and glycolytic rates were determined. Following basal measurements, cells were treated with oligomycin (2.5 μM), carbonyl cyanide 4-(trifluoromethoxy) phenylhydrazone (FCCP, 0.5 μM; Sigma C2920) and Antimycin (2 μM), rotenone (2 μM).

### Metabolomics

Mass spectrometry experiments were performed in the Northwest Metabolomics Research Center at the University of Washington, using the broad-based LC-MS/MS approach[37–40]. 5–8 mg samples of frozen kidney was collected from age and matched *Fnip1*^*-/-*^ and *Fnip1*^*+/-*^ control mice were homogenized in 50% methanol at ^-^80°C (n = 4/group). Results were normalized based on tissue weight. Mass spectrometry was performed as previously described[12]. Data were analyzed using MetaboAnalyst 3.0. Partial Least Squares-Discriminant Analysis (PLS-DA), which uses multivariate regression techniques to extract linear combination of variables, was utilized to establish differences. Variable Importance in Projection (VIP) is the weighted sum of squares of the PLS loadings taking into account the amount of explained Y-variation in each dimension[41].

### RNAseq

RNA was purified from frozen kidney tissue with the use of RNeasy Mini Kit (Qiagen Valenica, CA). Total RNA integrity was checked using an Agilent 2200 TapeStation (Agilent Technologies, Inc., Santa Clara, CA) and quantified using a Trinean DropSense96 spectrophotometer (Caliper Life Sciences, Hopkinton, MA). RNA-seq libraries were prepared from total RNA using the TruSeq RNA Sample Prep Kit (Illumina, Inc., San Diego, CA, USA) and a Sciclone NGSx Workstation (PerkinElmer, Waltham, MA, USA). Library size distributions were validated using an Agilent 2200 TapeStation (Agilent Technologies, Santa Clara, CA, USA). Additional library QC, blending of pooled indexed libraries, and cluster optimization were performed using Life Technologies’ Invitrogen Qubit^®^ 2.0 Fluorometer (Life Technologies-Invitrogen, Carlsbad, CA, USA).

RNA-seq libraries were pooled (6-plex) and clustered onto a flow cell lane. Sequencing was performed using an Illumina HiSeq 2500 in rapid mode employing a paired-end, 50 base read length (PE50) sequencing strategy. Image analysis and base calling were performed using Illumina’s Real Time Analysis v1.18 software, followed by ‘demultiplexing’ of indexed reads and generation of FASTQ files, using Illumina’s bcl2fastq Conversion Software v1.8.4 (http://support.illumina.com/downloads/bcl2fastq_conversion_software_184.html).

For RNA-seq data Analysis, reads of low quality were filtered prior to alignment to the reference genome (UCSC mm10 assembly) using TopHat v2.1.0[42]. Counts were generated from TopHat alignments for each gene using the Python package HTSeq v0.6.1[43]. Genes with low counts in greater than 2 samples were removed, prior to identification of differentially expressed genes using the Bioconductor package edgeR v3.12.0[44]. A false discovery rate (FDR) method was employed to correct for multiple testing[45]. Differential expression was defined as log_2_ (ratio) | ≥ 1 (± 2-fold) with the FDR set to 5%.

### Transmission electron microscopy

Kidneys were harvested from 8 week-old *Fnip1*^*-/-*^ and *Fnip1*^*+/-*^ (wildtype) mice were chopped into ~ 1 mm^3^ pieces and preserved in Karnovsky’s Gluteraldehyde fixative. Samples were dehydrated, sectioned into 70-100nm sections at the Vision Core Lab of the University of Washington (Seattle, WA). Tissue slices were epoxy embedded and visualized with the use of a JEOL 1230 transmission electron microscope (JEOL Ltd., Tokyo, JAPAN). 10 images were taken from low and high power fields per mouse. Percent mitochondrial area was calculated using ImageJ (National Institutes of Health) (17). The ratio of mitochondrial area/total area was determined for n = 4 mice per genotype (3 high quality low power fields (images) per mouse).

#### Flow cytometry

Mice were perfused with saline by gravity force following CO_2_ euthanasia. Kidney tissues were harvested, diced with scissors into small pieces (~1mm diameter), and incubated with collagenase solution at 37 degrees for 30 minutes. Tissues were further dissociated using a syringe plunger and passed through 70μm cell strainer. Collected cells were lysed with ACK lysis buffer (to remove all red blood cell contamination), and stained with fluorescent-conjugated antibodies specific against CD3, B220, Gr1 [18]. Flow cytometric data were acquired on a FACSCanto II or LSRII flow cytometer (BD Biosciences), and data were analyzed using FlowJo software.

### Statistics

Data were analyzed with the Student’s two-tailed *t*-test. Kaplan-Meier curve statistics were performed with Log-rank Mantel-Cox test. Seahorse data were analyzed by paired *t*-test of the mean values for each time-point. For real-time quantitative PCR, differences were calculated using the comparative C_T_ method[36]. p< 0.05 were considered significant.

## Results

### Disruption of *Fnip1* results in increased kidney weight and renal cyst formation

**Fig 1:**
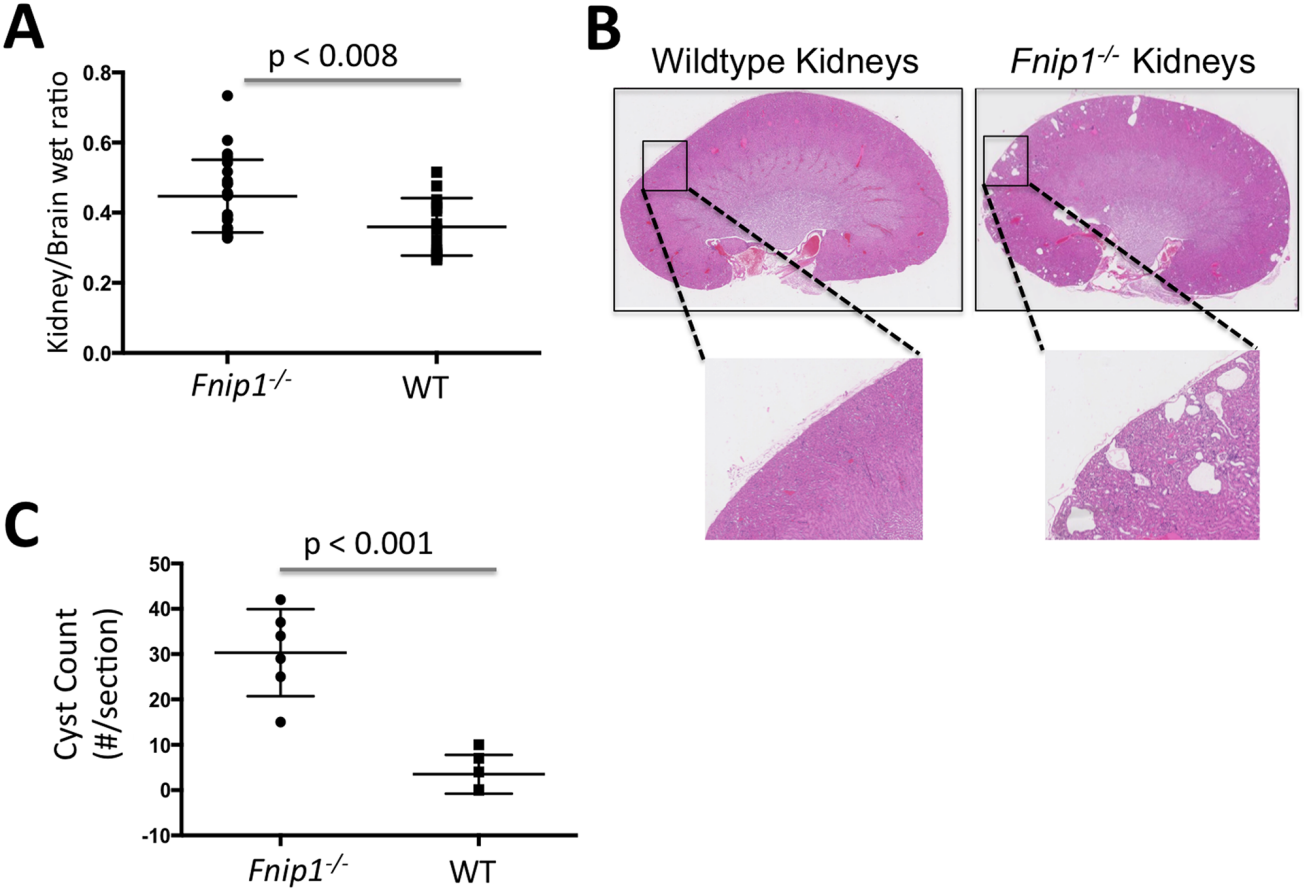
Loss of Fnip1 results in increased kidney size and cyst formation. (A) Increased kidney-to-brain weight ratio in *Fnip1*^*-/-*^ versus wildtype mice. n = 15 mice per genotype. Shown are means +/- SEM. (B) Representative hematoxylin and eosin stained histology sections of *Fnip1*^*-/-*^ and wildtype kidneys. *Fnip1*^*-/-*^ mice develop cysts and microcysts in the renal cortex. (C) Graph showing cyst counts, as determined by a blinded counter, of 12–18 week-old *Fnip1*^*-/-*^ and WT mice (n = 6 per group). *p-values are shown.

Because inactivating mutations in the *BHD* gene encoding Folliculin result in renal cancer, we sought to determine if loss of *Fnip1* alters kidney development and/or function in *Fnip1*-null mice, which were previously generated using ENU chemical mutagenesis [18]. We first assessed kidney size by measuring kidney-to-brain weight ratios following disruption of *Fnip1*. We found that kidney-to-brain weight ratios were significantly greater in *Fnip1*^*-/-*^ mice (Fig 1A) compared to wildtype (WT) mice, indicating that loss of *Fnip1* results in increased kidney weight. Analyses of hematoxylin and eosin (H&E) stained histologic sections of kidney revealed that there were increased numbers of cysts (≥ 5X normal tubule diameter) and microcysts (<5x normal tubule diameter) in the renal cortex of *Fnip1*^*-/-*^ mice compared to WT mice (Fig 1B and 1C). The cysts and microcysts (hereafter collectively called cysts) were lined by renal tubular epithelium, which tended to form blebs into the cyst lumen. Loss of *Fnip1* did not change urine specific gravity relative to WT mice, suggesting that renal function was not perturbed following disruption of *Fnip1* (data not shown). These results collectively suggest that loss of *Fnip1* alters renal morphology, leading to the formation of cysts in the renal cortex.

### *Fnip1* loss results in decreased AMPK activation and increased mTORC1 activity

**Fig 2:**
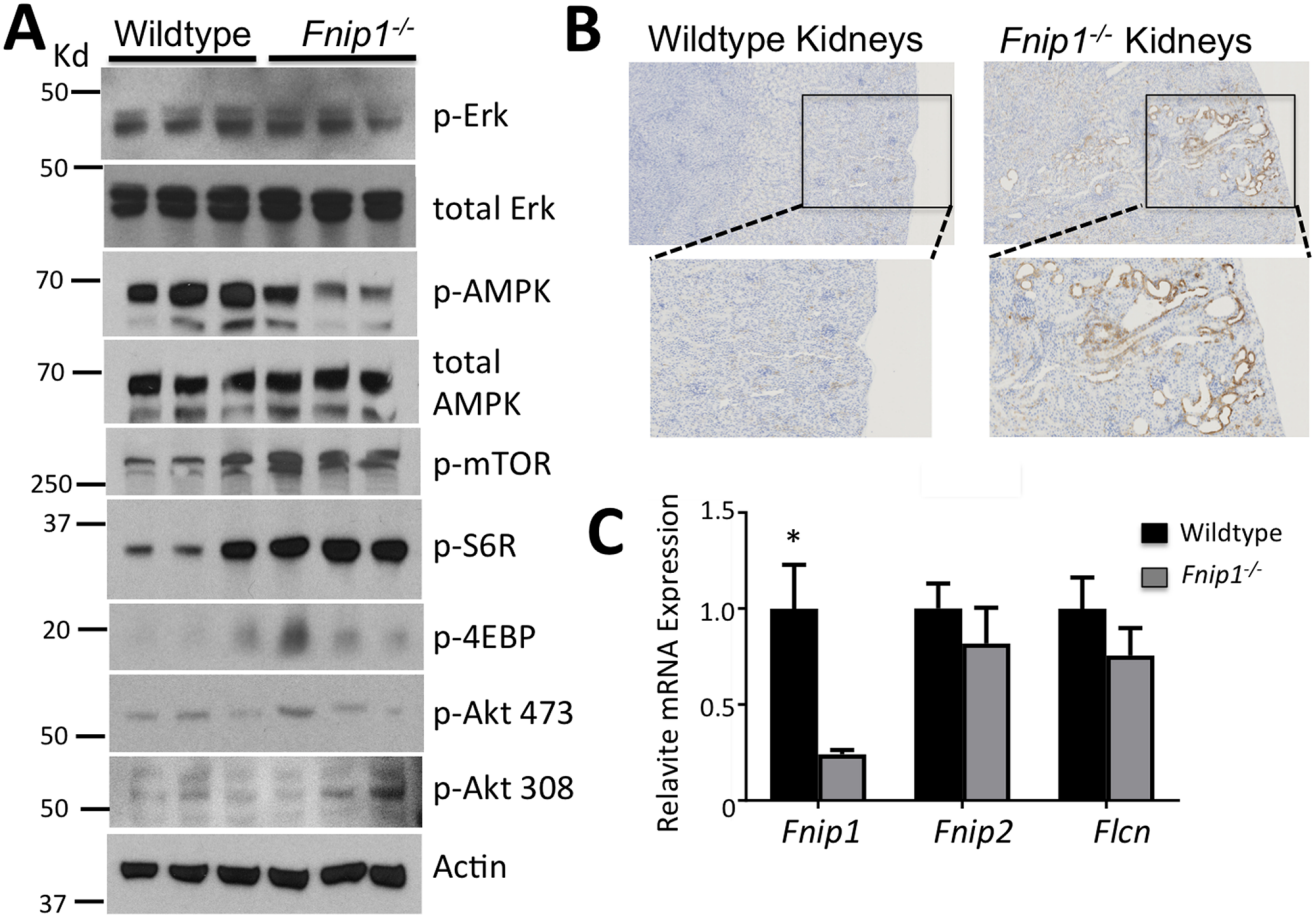
Increased mTOR and decreased AMPK activation in *Fnip1* null kidney tissue and renal tubular epithelial cells. (A) Representative immunoblots showing decreased AMPK activation (pAMPKThr172) and increased mTORC1 activation (p-S6R, p-4EBP1) in total renal tissue from *Fnip1*^*-/-*^ and WT mice. Statistical quantitation is shown in S1 Fig. n = 3 mice per genotype (each lane). (B) Representative immunohistochemistry images showing increased p-S6R activation around cystic tubules in *Fnip1*^*-/-*^ mice. Representative of 3 mice per genotype. (C) Real-time quantitative PCR showing no significant changes in expression of *Flcn (Bhd)* and *Fnip2* mRNA in *Fnip1*^*-/-*^ renal tissue versus WT (n = 3/genotype, * = p = 0.03). Mice used were between 12–18 weeks of age.

Disruption of the *Bhd* gene in mice results in polycystic kidney disease (PKD) characterized by altered mTORC1 activation. To determine why loss of *Fnip1* could result in increased renal weight and cyst formation, we measured activation of AMPK and the mTOR pathways by immunoblotting and immunohistochemistry (IHC). Immunoblotting of whole kidney lysate from *Fnip1*^*-/-*^ and WT mice revealed decreased phosphorylation of AMPK at threonine 172 indicative of decreased AMPK activation, increased phosphorylation of mTOR, and increased phosphorylation of S6 ribosomal protein (S6R), a downstream target of mTORC1 activation (Fig 2A and S1 Fig). Phosphorylation of Erk1/2, Akt^Thr308^, and Akt^Ser473^ were not significantly different in *Fnip1*^*-/-*^ versus WT cells, suggesting no significant effect of *Fnip1* loss on Erk, PI3K, or mTORC2 signaling. Using IHC, we found local increases in p-S6R in the renal tubular epithelial (RTE) cells lining the cysts in the *Fnip1*^*-/-*^ kidneys (Fig 2B). mRNA expression of *Fnip2* and *Bhd* (Flcn) were unchanged (Fig 2C) in *Fnip1* null kidneys, indicating that the phenotypes are not due to secondary alterations in their expression. These results indicate that loss of *Fnip1* results in decreased AMPK activation and increased mTORC1 activation in RTE cells.

### *Fnip1* deficient renal epithelial cells exhibit increased oxidative phosphorylation

**Fig 3:**
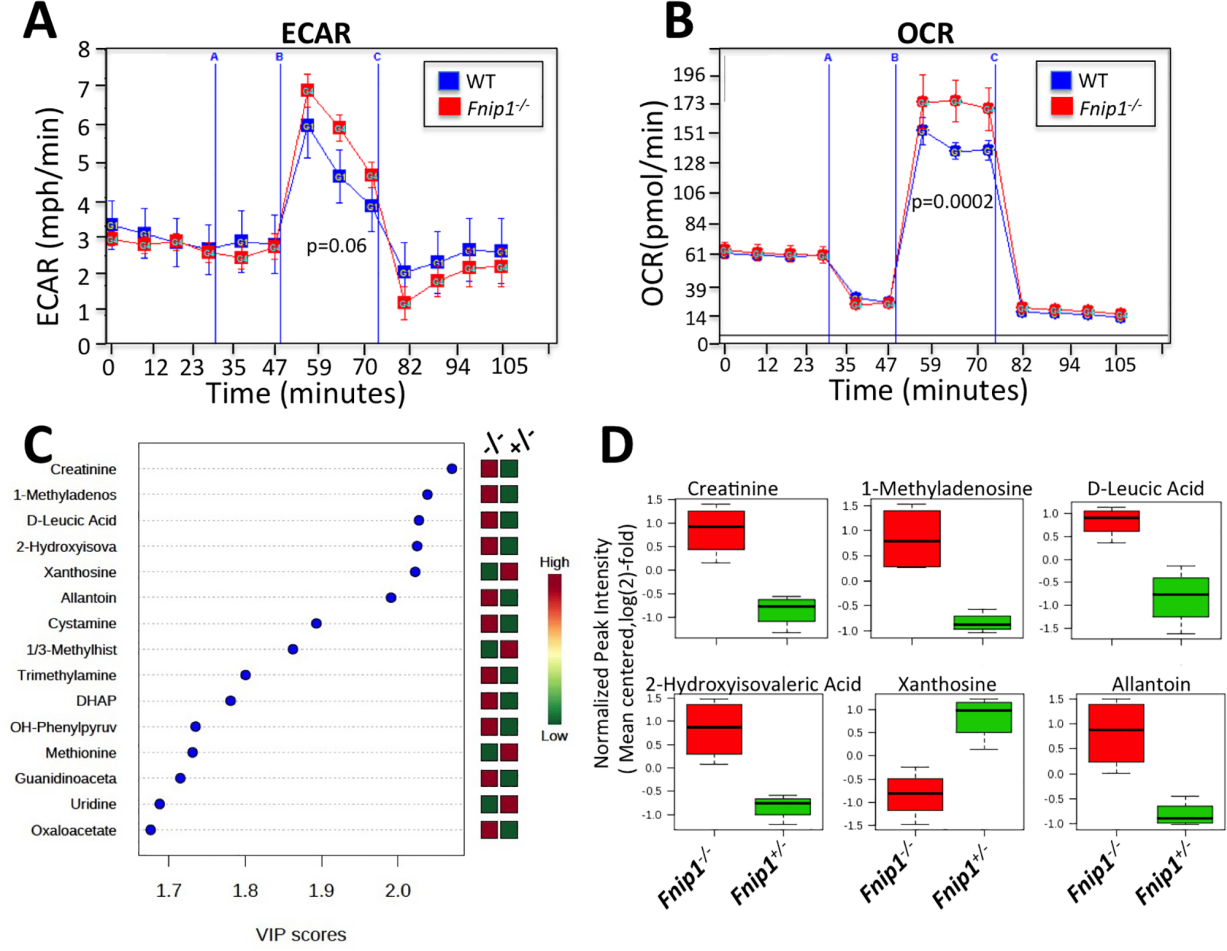
*Fnip1*^*-/-*^ kidney tubular epithelial cells exhibit increased metabolism. (A) Seahorse analyses showing increased maximal extracellular acidification rate (ECAR), a measure of glycolysis, and (B) increased maximal oxidative phosphorylation (OCR) of cultured purified *Fnip1*^*-/-*^ (n = 3 mice) versus WT (n = 3 mice) renal tubular epithelial cells. Cells were stimulated with oligomycin (2.5 μM) at 30 minutes, FCCP (0.5 μM) at 50 minutes, and rotenone (2 μM) and antimycin A (2 μM) at 75 minutes. p-values are shown. (C and D) Total kidneys from *Fnip1*^*-/-*^ (n = 4) and WT (n = 3) mice (20–32 weeks old) were analyzed by liquid chromatography tandem mass spectrometry (LC-MS/MS) to determine metabolite levels. Data were analyzed using MetaboAnalyst 3.0. Variable importance in projection (VIP) scores of metabolite changes in *Fnip1*^*-/-*^ versus wildtype renal tissue showing changes in nitrogenous waste products, amino acid metabolites, glycolysis, and TCA cycle metabolites. Metabolites that were significantly different and either increased (red) or decreased (green) are shown. (D) Whisker plots showing selected metabolites either significantly increased or decreased in *Fnip1*^*-/-*^ (red) versus *Fnip1*^*+/-*^(green) kidney tissue. p-values are listed in S1 Table.

Both PKD and renal cancer exhibit metabolic changes characterized by increased glycolysis and/or oxidative phosphorylation. We next assessed whether loss of *Fnip1* and subsequent mTORC1 activation altered the metabolic characteristics of *Fnip1* null renal tubular epithelial cells. RTE cells were isolated from *Fnip1*^*-/-*^ and WT mice, and basal metabolic characteristics were measured using the Seahorse XF analyzer, which measures oxygen consumption rate (OCR; a measure of oxidative phosphorylation) and extracellular acidification rate (ECAR; a measure of glycolysis). *Fnip1*^*-/-*^ RTE cells exhibited increased maximal OCR (Fig 3B) relative to WT renal tubular epithelial cells following FCCP (carboxy cyanide-4 trifluromethoxy phenylhydrazone) stimulation (a mitochondrial membrane uncoupler), indicating that loss of *Fnip1* results in increased maximal respiration. Although not significantly different (p = 0.06), there was a trend towards increased glycolysis following *Fnip1* loss.

In order to better define this observed metabolic shift, we took an unbiased broad-based metabolomic approach using liquid chromatography tandem mass spectrometry (LC-MS/MS) to measure alterations in levels of metabolites associated with major metabolic pathways. We found that *Fnip1*^*-/-*^ kidneys contained increased levels of metabolites derived from the amino acid leucine (D-Leucic acid, 2-hydroxyisovaleric acid, and OH-phenylpyruvate), a major inducer of mTORC1 activation, relative to WT kidneys (Fig 3C and 3D and S1 Table). *Fnip1*^*-/-*^ kidneys also exhibited increased levels of glycolysis and TCA cycle metabolites including dihydroxyacetone phosphate (DHAP) and oxaloacetate respectively. Increases were also seen in the purine metabolites 1-methyladenosine and allantoin (Fig 3C and 3D and S1 Table), consistent with increased purine biosynthesis in *Fnip1*^*-/-*^ kidneys. Interestingly, 1-methyladenosine is elevated in the urine of patients with malignant cancers [46, 47], and promotes translation of methylated mRNAs leading to increased protein production [48]. These results support increased metabolic activity following disruption of *Fnip1* (Fig 3), and are also consistent with increased mitochondrial biogenesis, oxidative phosphorylation, glycolysis, and purine biosynthesis (see [49] for review).

### Global gene expression analyses reveal alterations in ion transporters, cell adhesion molecules, and immune cell infiltration

**Fig 4:**
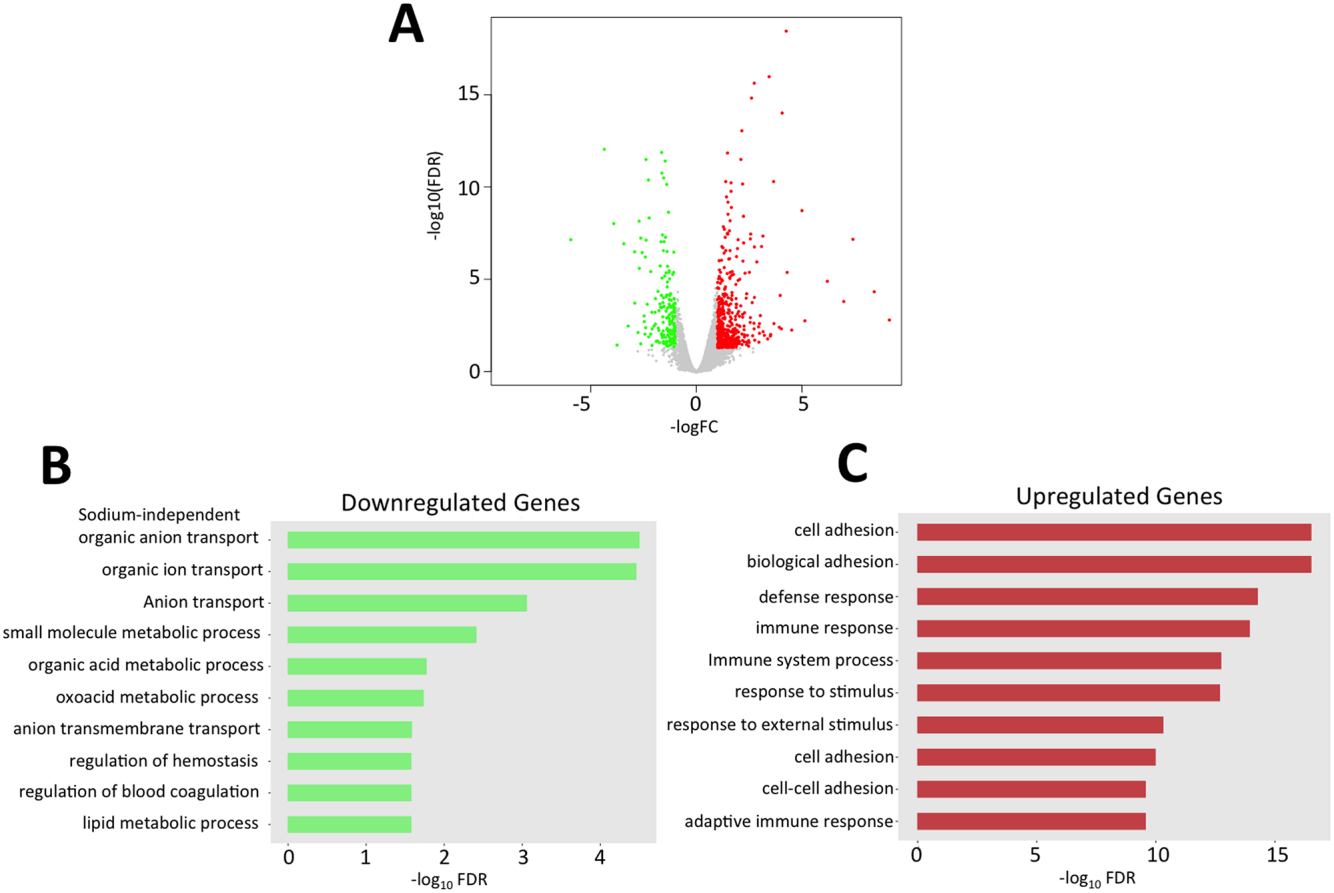
Differences in gene expression between *Fnip1*^*-/-*^ versus wildtype kidney tissue. RNAseq was performed on kidney cortical tissue from *Fnip1*^*-/-*^ (n = 3) and wildtype (n = 3) mice (11–18 weeks of age) using Illumina HiSeq 2500. (A) Volcano plot generated using EdgeR program showing genes significantly increased (red) and decreased (green) in *Fnip1*^*-/-*^ kidney versus WT kidney tissue. Gene ontology analyses derived from Bioconductor Open Source Software for Bioinformatics showing: (B) significantly downregulated genes grouped by expression levels and gene function in *Fnip1*^*-/-*^ compared to WT renal tissue, and (C) significantly upregulated expression of genes grouped by expression levels and gene function in *Fnip1*^*-/-*^ compared to WT renal tissue. The majority of significantly decreased genes were involved in ion transport and metabolism, whereas the majority of significantly increased genes were involved in cell adhesion and immune responses.

To assess global mRNA changes associated with disruption of *Fnip1* in renal tissue, we performed RNAseq on cortical kidney tissue from *Fnip1*^*-/-*^ and *Fnip1*^*+/-*^ mice, followed by quantitative PCR to assess changes in gene expression of selected genes of interest. We found that mRNA expression of 651 genes were significantly different in *Fnip1*^*-/-*^ kidney tissue versus *Fnip1*^*+/-*^ tissue. Of the 651 genes, expression of 445 genes were upregulated and 206 genes were downregulated following disruption of *Fnip1*. Kyoto Encyclopedia of Genes and Genome (KEGG) analyses indicated that of the downregulated genes, there was significant enrichment for genes associated with sodium independent ion transport, organic ion transport, anion transmembrane transport, and lipid metabolism (Fig 4A and 4B and S2 Table). In contrast, of the upregulated genes, there was significant enrichment in expression of genes associated with cell adhesion and immune response (Fig 4C and S2 Table). While it is formally possible that some of these mRNA changes may reflect differences in the representation of specific cell types in cortical tissue from *Fnip1*^*+/-*^ versus *Fnip1*^*-/-*^, the vast majority of RNA is derived from similar renal cortical cell types (tubular epithelial cells, glomerulus parietal cells, podocytes, etc) which likely reflect the majority of transcriptional differences.

**Fig 5:**
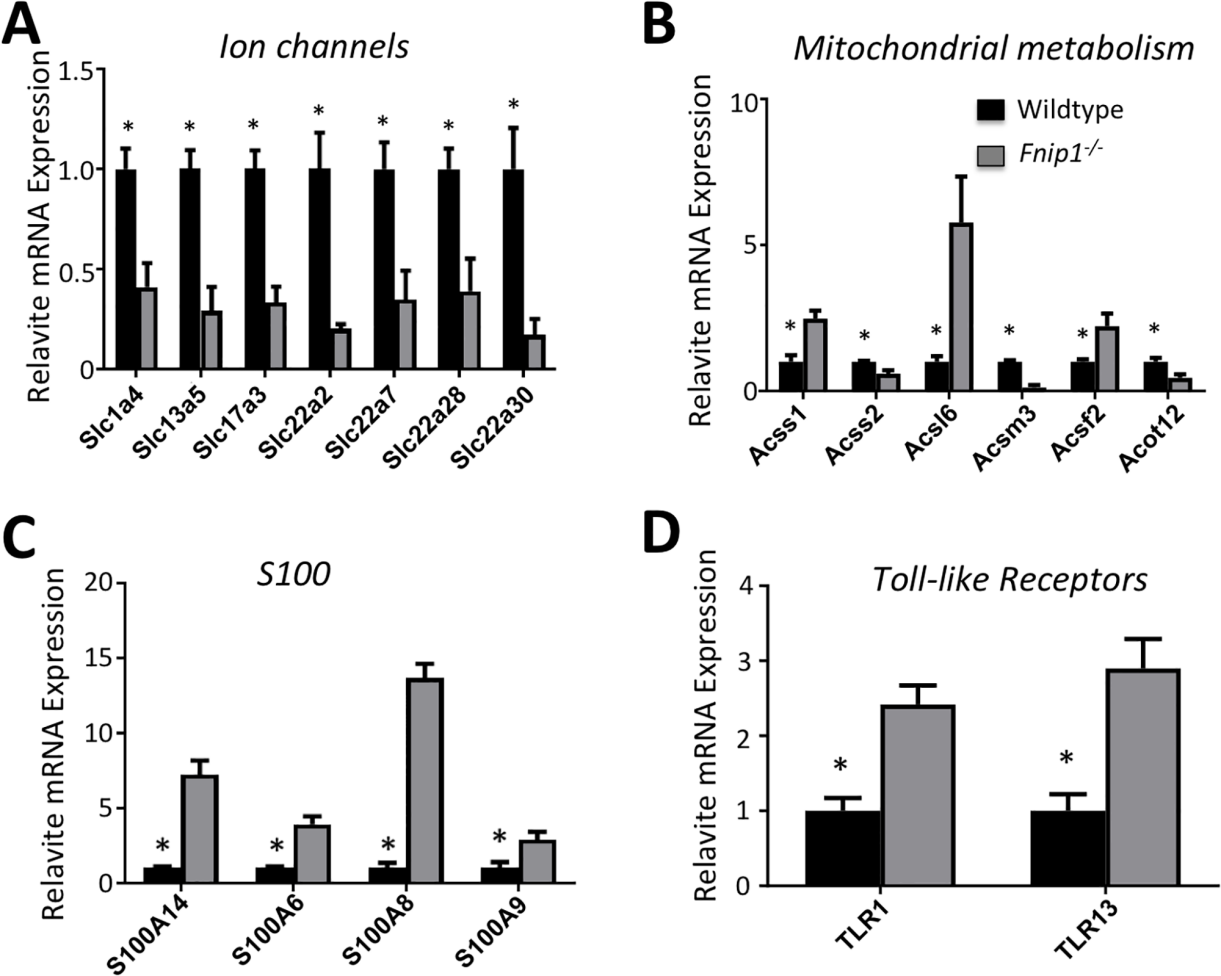
Real-time Quantitiative PCR analyses on kidney tissue from *Fnip1*^*-/-*^ versus wildtype mice. RNA was purified from *Fnip1*^*-/-*^ (n = 3) compared to WT (n = 3) kidney cortical tissue (mice were 11–18 weeks of age). Quantitative PCR was performed on cDNA derived from these tissues. Bar graphs show fold- differences in gene expression in *Fnip1*^*-/-*^ compared to WT mice (where WT is set to 1). (A) Genes encoding many of the solute carrier (Slc) family of transporter proteins are decreased in *Fnip1*^*-/-*^ compared to WT kidney tissue. (B) Altered expression of genes encoding acyl-CoA regulatory molecules and fatty acids regulatory molecules. (C) Expression of S100A genes including A14, 6, 8, and 9 are increased in *Fnip1*^*-/-*^ compared to WT kidney tissue. (D) Increased expression of TLR1 and 13 genes in *Fnip1*^*-/-*^ compared to WT kidney tissue. (n = 3 of each genotype, * = p<0.01).

Quantitative PCR analyses confirmed that expression of many *Slc* family of transporter genes were downregulated following disruption of *Fnip1* (S2 Table and Fig 5A). For example, expression of *Slc1a4* and *Slc17a3*, which are involved with glutamate transport, were both significantly decreased in *Fnip1*^*-/-*^ versus *Fnip1*^*+/-*^ tissue. Similarly, expression of *Slc13a5*, and *Slc24a3*, genes involved with Na^+^ transport, were also decreased in *Fnip1*^*-/-*^ versus *Fnip1*^*+/-*^ tissue. Genes important for the transport of organic molecules in the proximal renal tubule were also decreased including *Slc22a2*, which resorbs organic cations; *Slc22a6* and *Slc22*a7, which are involved with the transport of anions; and *Slc22a12*, which transports urate for excretion and is important for proper kidney function. Of the *Slc* genes with increased expression, *Slc15a3* is a proton/oligopeptide cotransporter, and *Slc22a3* is an organic cation transporter that is expressed in several tissues including liver, intestine, nervous, and kidney. These results collectively suggest that loss of *Fnip1* in renal tissue results in significant alterations in the expression of an array of integral membrane ion transporters.

Our Seahorse and metabolomics analyses suggested that mitochondrial metabolism is increased in *Fnip1* null tissue. Consistent with this notion, RNAseq and quantitative PCR data showed changes in multiple genes involved in Acyl-CoA pathway and fatty acid metabolism (Figs 5B and S2). For example, expression of *Acox3*, *Acsm 3*, and *CD36*, which are all involved with fatty acid synthesis, were decreased in *Fnip1*^*-/-*^ mice, whereas expression of genes associated with fatty acid binding and shuttling were increased, including *Fabp1*, *Fabp4*, *Perilipin1*, and *Perilipin 4* (S2 Table and Fig 5). Expression of *Acss1* (acetyl-CoA synthetase), which is important in the TCA cycle was increased, while expression of *Acot12*, which hydrolyzes Acetyl CoA, was decreased. These increases in metabolites and the changes seen in expression of Acyl-CoA genes are consistent with increased mitochondrial metabolism. To determine whether mitochondrial density or morphology was altered following disruption of *Fnip1*, we performed electron microscopy on renal tissue from *Fnip1*^*-/-*^ and WT mice. Although no statistically significant changes were noted, there was a trend towards increased mitochondrial area in *Fnip1*^*-/-*^ RTE cells relative to WT cells (S2 Fig).

To define how loss of *Fnip1* alters mRNA expression, we utilized the Ingenuity Upstream Regulator Analyses program to help illuminate the cascade of transcriptional regulators that are differentially regulated in the RNAseq data derived from *Fnip1*^*-/-*^ vs wildtype kidney tissue[50]. Interestingly, we found that loss of Fnip1 resulted in the most significant enrichment for Myc-regulated genes, followed by HDAC1, HDAC2, FoxA1, and HoxA10, which were predicted to be activated based on the direction of the gene expression change following disruption of *Fnip1* (S3 Table). Other transcriptional regulators with significant enrichment included Spi1, Ets1, Creb, HTT, PGC1alpha, and TP63. Although Ingenuity was unable to predict activation versus repression for PGC1alpha or Myc in our RNAseq study (likely because the program does not take into account the presence or absence of co-activators, co-repressors, or dominating repressive complexes), increased Myc and PGC1alpha expression have been associated with PKD and renal cancer [17, 51, 52].

Recent studies suggest that increased kidney inflammation is associated with PKD in humans[53, 54]. RNAseq and quantitative PCR revealed that genes involved in immunity and inflammation were increased in *Fnip1*^*-/-*^ renal tissue relative to WT renal tissue, including the S100, Toll-like receptors (TLR), NOD-like receptors, FC-receptors, MHC, and genes encoding complement (Fig 5C and 5D and S2 Table). Specifically, *Fnip1*^*-/-*^ renal tissue was characterized by increased expression of complement components including component 3 (*C3* and *C3ar1*), which are important in both the classical and alternate pathways of complement signaling, and *C1qa*, *b*, and *c*, which are all part of the *C1q* complex that is important in the classical pathway (S2 Table). Similarly, increases were seen in the expression of the *S100A14*, *S100A6*, *S100A8*, and *S100A9* pro-inflammatory genes (Fig 5C and S2 Table). S100A14 affects the p53 pathway and modulates expression of matrix metalloproteases MMP1 and MMP9. S100A6 functions in in cell proliferation, cytoskeletal dynamics, and tumorigenesis, thus promoting cell division and tumor growth. S100A8 is highly expressed in the cytosol of some immune cells including neutrophils, macrophages, and dendritic cells, and is induced by toll-like receptor (TLR) agonists, and S100A9 inhibits myeloid differentiation thereby contributing to tumor growth. We also found that expression of *Tlr1*, *Tlr7*, and *Tlr13* were also increased in *Fnip1*^*-/-*^ versus WT kidney tissue (Fig 5D and S2 Table). Toll-like receptors are important for activation of the innate immune system by recognizing pathogen-associated molecular patterns in microbes.

**Fig 6:**
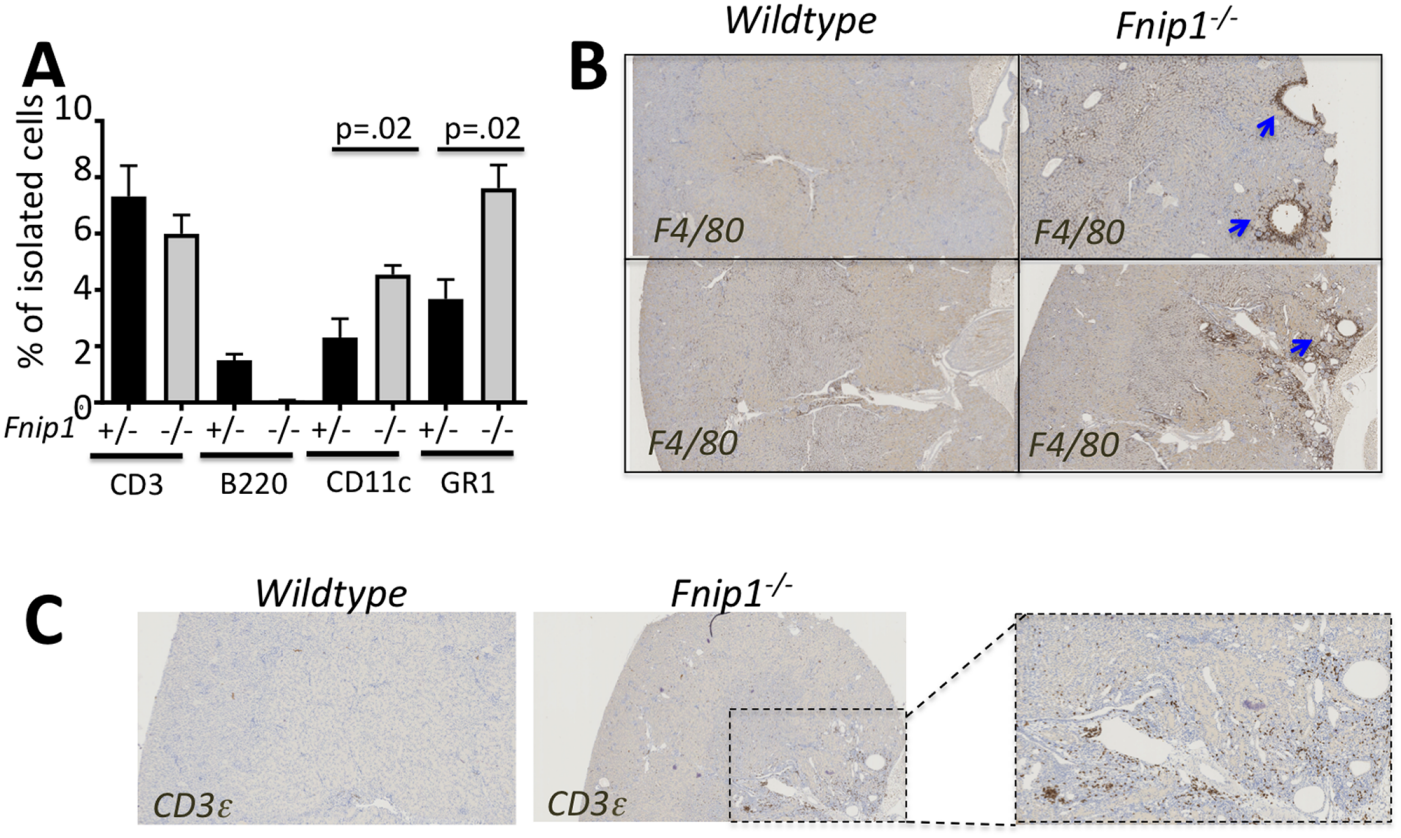
Increased immune cell infiltration in *Fnip1*^*-/-*^ null kidney tissue. (A) Flow cytometric analyses of immune cells isolated from *Fnip1*^*-/-*^ and wildtype kidney tissue showing increases in the representation of CD11c and GR1 labeled cells and decreases in B220 labeled cells (n = 3 of each genotype, p-values are shown). (B) Representative immunohistochemistry images showing increased representation of F4/80^+^ myeloid cells and (C) increased representation of CD3^+^ cells localized around renal cysts of *Fnip1*^*-/-*^ mice (arrows). Micrographs are representative of 3 mice per group (~12 weeks of age).

To further determine whether disruption of *Fnip1* was associated with increased inflammation in *Fnip1* null renal tissue, we purified immune cells from *Fnip1*^*-/-*^ and *Fnip1*^*+/-*^ kidney tissue and defined the representation of specific immunocytes by flow cytometry. Compared to *Fnip1*^*+/-*^ renal tissue, *Fnip1*^*-/-*^ renal tissues exhibited a significant increase in the representation of CD11c^+^ and GR1^+^ myeloid cells (Fig 6A), whereas the percentages of total CD3^+^ T cells were not significantly different. B220^+^ B cells were decreased following disruption of *Fnip1*, due to a block in B cell development[18]. CD11c is an integrin expressed on monocytes, macrophages, and neutrophils, and GR1 protein is predominantly expressed on bone marrow and peripheral blood neutrophils. Interestingly, using IHC we found focal increases in F4/80 staining localized around the cysts in sections of kidney from *Fnip1*^*-/-*^ relative to WT mice (Fig 6B). The F4/80 antigen is a glycoprotein that is expressed by murine macrophages. In contrast to our data on the representation of T cells in total renal tissue, we found focal increases in CD3epsilon positive T cells around renal cysts as well (Fig 6C). These results suggest that disruption of *Fnip1* results in increased representation of inflammatory cells infiltrating *Fnip1* deficient kidney tissue, which appears to be localized around renal cysts.

### Fnip1 and Tsc1 synergize to inhibit mTORC1 activation and polycystic kidney disease

**Fig 7:**
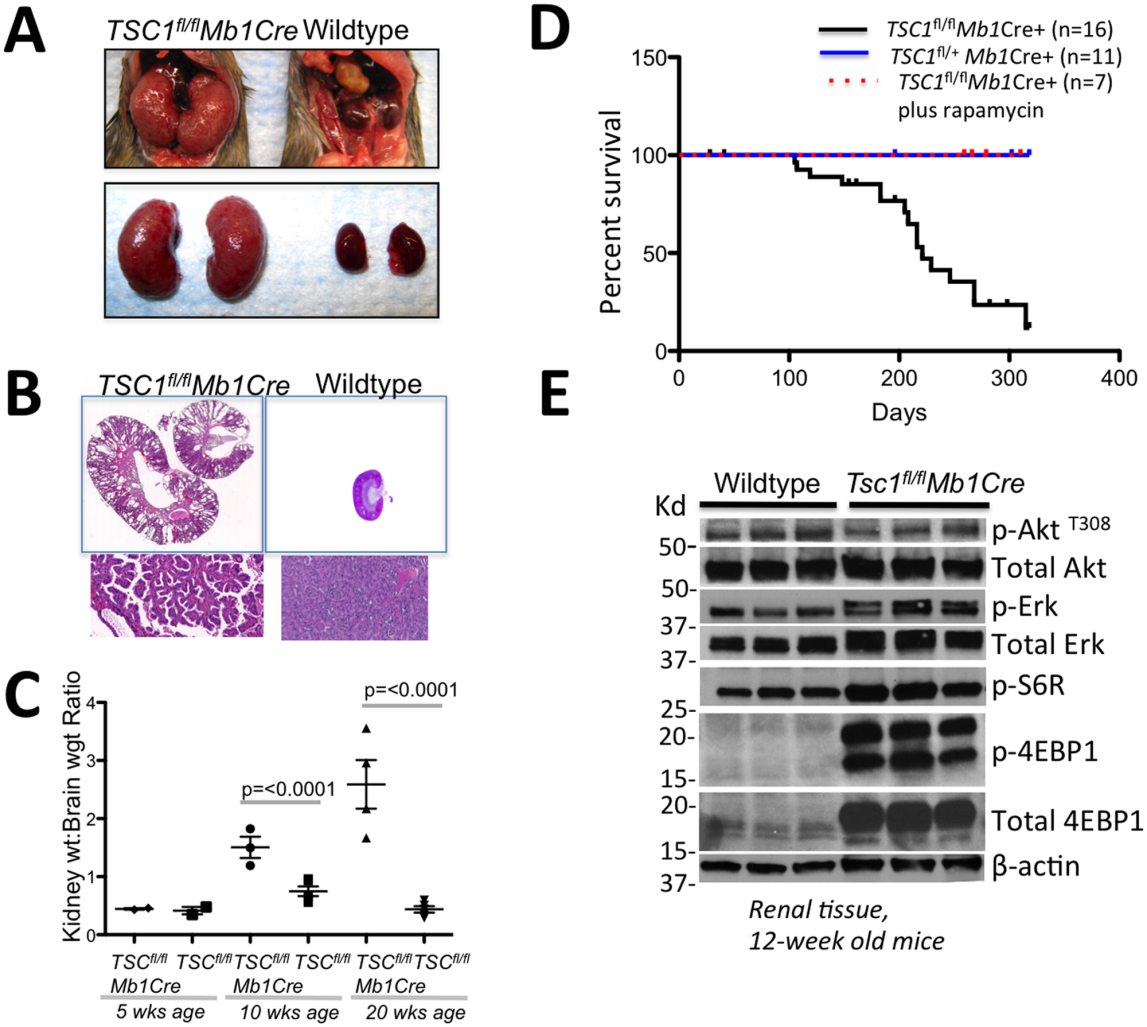
Conditional disruption of *Tsc1* results in polycystic kidney disease. *Tsc1*^*fl/fl*^ mice were bred to *Mb1Cre*^+^ mice. (A) Representative gross pictures of *Tsc1*^*fl/fl*^ *Mb1Cre*^+^ and WT kidneys showing that loss of *Tsc1* results in kidney enlargement in 20-week old mice. (B) Representative histology images of *Tsc1*^*fl/fl*^ *Mb1Cre*^+^ kidneys showing development of polycystic kidney disease at 20 weeks of age. (C) Kidney-to-brain ratio graph showing ratios at 5, 10, and 20 week old *Tsc1*^*fl/fl*^ *Mb1Cre*^+^ and WT mice showing a gradual increase in kidney-to-brain weight ratio in *Tsc1*^*fl/fl*^ *Mb1Cre*^+^ kidneys. (n = 3 each genotype, p-values are shown) (D) Survival graph of *Tsc1*^*fl/fl*^ *Mb1Cre*^+^ (solid black line), *Tsc1*^*fl/+*^ *Mb1Cre*+ (sold blue line), and rapamycin treated *Tsc1*^*fl/fl*^ *Mb1Cre*^+^ (dotted red line) showing that polycystic kidney disease is fatal, but that disease induction can be blocked by inhibiting mTOR with Rapamycin. Rapamycin diet was started at weaning and continued until euthanasia. (E) Immunoblots of proteins isolated from whole kidney lysates from 12-week old mice showing increased p-S6R and p-4EBP indicative of increased mTORC1 activation, and increased p-Erk in *Tsc1*^*fl/fl*^ *Mb1Cre*^+^ relative to WT kidney tissue.

Kidney tissues from human patients with BHDS, and murine kidney tissue from mice with gene-targeted mutations in *Bhd*, are generally characterized by increased mTORC1 activation. However, recent studies whereby *BHD*, *FNIP1*, or *FNIP2* were knocked down in cell lines suggested that the Folliculin/Fnip1/Fnip2 complex may be required to activate mTORC1[24, 25]. To further assess how disruption of *Fnip1* modifies mTORC1 signaling and PKD in vivo, we generated a murine model of PKD, whereby the mTORC1 inhibitor *Tsc1* gene was disrupted using *Mb1*Cre, which is predominantly expressed in hematopoietic cells and to a lesser extent in kidney[32]. We then bred *Fnip1*^*-/-*^ mice to *Tsc1*^*fl/fl*^*Mb1*Cre mice to define the consequences of *Fnip1* loss on hyperactive mTOR signaling and the development of PKD driven by *Tsc1* loss. *Tsc1*^*fl/fl*^*Mb1*Cre mice alone developed significant PKD by 10 weeks of age (Fig 7A and 7B) characterized by a ~ 2-fold increase in kidney/brain weight ratio and increased cystic structures at 10 weeks of age, which progressed to a ~5-fold increase in kidney/brain weight by 20 weeks of age (Fig 7C). *Tsc1*^*fl/fl*^*Mb1*Cre mice also exhibited decreased survival, which could be completely rescued by provision of a rapamycin diet (Fig 7D). Analyses of renal tissue from 12 week-old *Tsc1*^*fl/fl*^*Mb1*Cre and age-matched WT mice revealed significantly increased Erk-phosphorylation (Figs 7E and S4)). Phosphorylation of S6R and 4E-BP1, downstream targets of mTORC1 signaling, were also significantly increased in *Tsc1*^*fl/fl*^*Mb1*Cre mice relative to age matched wildtype mice.

**Fig 8:**
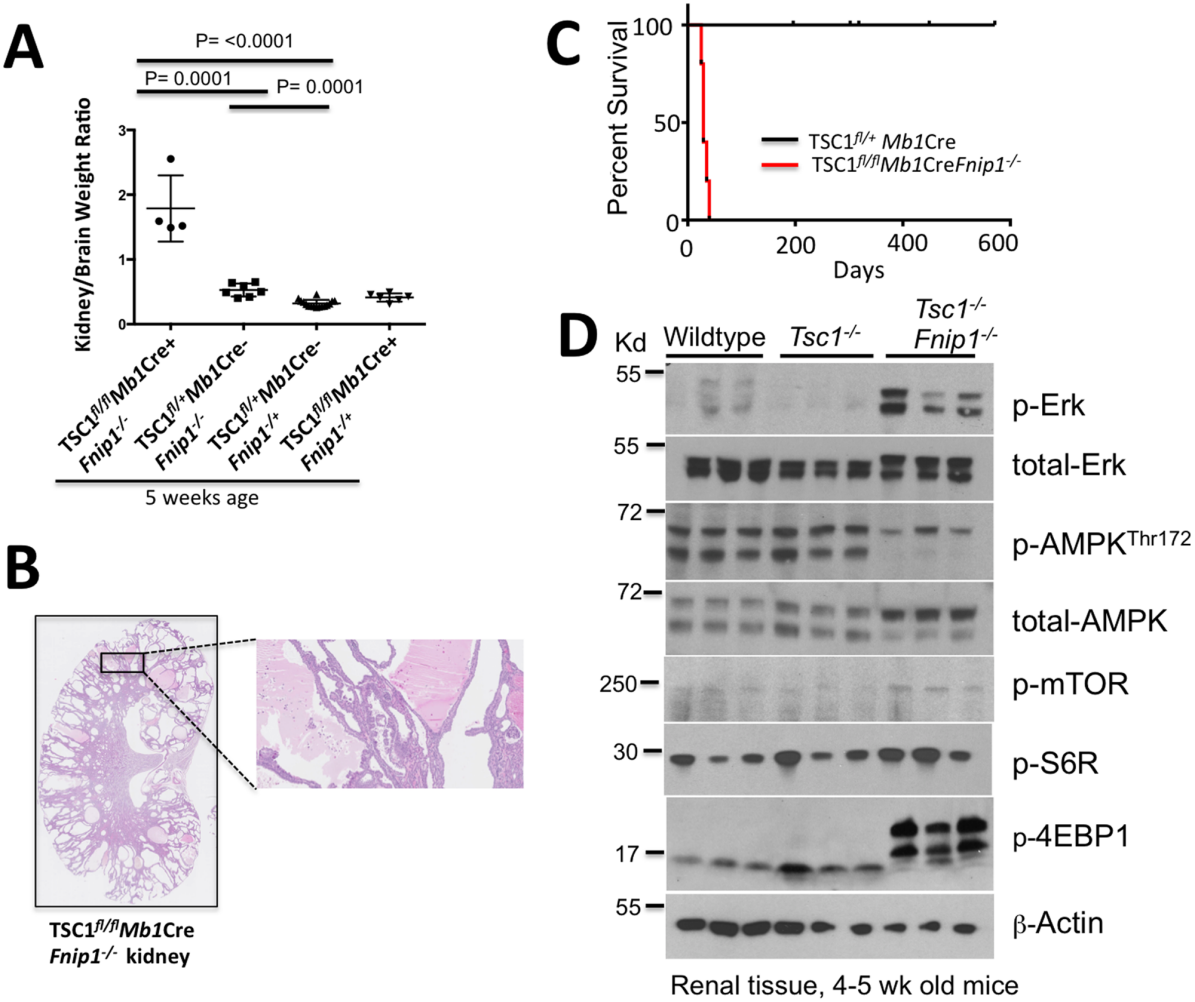
Loss of Fnip1 synergizes with loss of TSC1 resulting in accelerated PKD and mTOR activation. (A) Kidney-to-brain ratio graph comparing double null (*Tsc1*^*fl/fl*^*Mb1*Cre^+^*Fnip1*^*-/-*^), *Fnip1*^*-/-*^ (*Fnip1*^*-/-*^*Tsc1*^*fl/+*^ *Mb1*Cre-), *Tsc1* null (*Fnip1*^*-/+*^ *Tsc1*^*fl/fl*^*Mb1*Cre^+^), and heterozygous (*Fnip1*^*-/+*^ *Tsc1*^*fl/+*^ *Mb1Cre*^-^) mice at 5 weeks of age, showing an increase in kidney size of double null mice at an early age. (B) Representative histology image of *Tsc1*^*fl/fl*^ *Mb1*Cre^+^*Fnip1*^*-/-*^ double null mice showing the development of severe polycystic kidney disease at 5 weeks of age. (C) Kaplan Meier survival graph of *Tsc1*^*fl/fl*^*Mb1*Cre^+^*Fnip1*^*-/-*^ double null and *Tsc1*^*fl/+*^*Mb1*Cre^+^*Fnip1*^*-/-*^ mice. Double-null mice develop PKD very early in life, indicating that loss of *Fnip1* in *Tsc1* null mice greatly accelerates the onset of PKD relative to *Tsc1* loss alone. (D) Immunoblots of proteins isolated from whole kidney lysates derived from 5 week-old wildtype, *Tsc1*^*-/-*^, and *Tsc1*^*-/-*^*Fnip1*^*-/-*^ blots showing increased p-Erk, p-S6R, and p-4E-BP in *Tsc1*^*-/-*^ *Fnip1*^*-/-*^ kidney tissue compared to *Tsc1*^*-/-*^ and WT mice indicating hyperactivation of the mTORC1 and Erk pathways. p-AMPK is decreased in double null mice relative to *Tsc1*^*-/-*^ and WT mice, suggesting decreased AMPK activation. (n = 3 each genotype, p-values are shown).

To define how loss of *Fnip1* modulates activated mTOR signaling in *Tsc1* null mice, we measured kidney-to-brain weight ratios in *Fnip1*^*-/-*^*Tsc1*^*fl/fl*^*Mb1*Cre double-null mice (*Fnip1Tsc1* double-null) mice compared to *Tsc1*^*fl/fl*^ *Mb1-Cre*^+^ mice alone. *Fnip1Tsc1* double-null mice developed large cystic kidneys (kidney/brain ratio mean 1.79) by 4 weeks of age (Fig 8A and 8B) whereas *Tsc1* null mice had normal appearing kidneys at 4 weeks (Figs 8A and 7C), and did not develop cystic kidneys of comparable kidney/brain weight ratio as *Tsc1Fnip1* double null mice until ~10 weeks of age. While the *Tsc1* null mice survived an average of 24 weeks until euthanasia (Fig 7D), *Tsc1Fnip1* double null mice only survived to 3–5 weeks of age before euthanasia was required (Fig 8C). Immunoblot analysis revealed increased phosphorylated Erk and decreased phosphorylated AMPK^Thr172^ in *Tsc1Fnip1* double null kidneys relative to *Tsc1*^*-/-*^ and WT kidneys. mTORC1 activation, as measured by phosphorylation of mTOR, ribosomal S6 protein (S6R), and 4E-BP1 were increased in *Tsc1*^*-/-*^*Fnip1*^*-/-*^ double null mice relative to *Tsc1*^*-/-*^ and WT mice (Figs 8D and S4). Interestingly, phosphorylated EIF4E-binding protein 1 (p-4E-BP1) levels were approximately 10-fold higher than either *Tsc1*^*-/-*^ or WT kidneys. These results indicate that there is strong synergism between loss of *Fnip1* and loss of *Tsc1*, and suggest that loss of these pathways utilize divergent mechanisms to activate mTORC1 in vivo in the absence of transformation.

## Discussion

Although disruption of Folliculin is known to predispose to renal cancer in humans, and PKD and renal cancer in mice, a specific role for Fnip1 has not been previously demonstrated. In this study, we found that disruption of *Fnip1* alone results in renal cyst formation and was surprisingly sufficient to initiate many of the molecular and cellular changes implicated in the development of PKD in humans, including decreased expression of organic ion transporters; increased oxidative metabolism; increased cellular growth; increased expression of cell adhesion molecules; and increased immune cell infiltration in areas directly surrounding cysts. We also provide evidence that Fnip1 normally synergizes with TSC1 to limit mTORC1 activation in renal tissue, thus mitigating the development of PKD. These results provide support that Fnip1 regulates a large functional molecular network in renal epithelial cells, which when disrupted, contributes to PKD and cancer.

Many of the cellular and molecular features of PKD have been characterized, although the molecular mechanisms of how these alterations result in PKD are still unclear. However, changes in signaling pathways that result in increased renal tubular epithelial cell proliferation, including activation of the mTOR and Ca^2+^ pathways, are primary features. For example, autosomal dominant polycystic kidney disease (ADPKD) in humans is characterized by the development of fluid filled kidney cysts, which eventually destroy normal kidney function and progress to renal failure. ADPKD is caused by mutations in either the *PKD1* or *PKD2* genes, which encode Polycystin 1 (PC1) and Polycystin 2 (PC2) respectively (see [55] for review). PC1 is found in the primary renal cilia where it localizes to the plasma membrane and forms adhesion complexes in polarized epithelial cells. PC1 is primarily important for cell-cell and cell-matrix interactions. PC2 is also a transmembrane protein that co-localizes with PC1 in the primary cilium. However, a large portion of PC2 is also localized in the intracellular compartment where it functions as a calcium permeable non-selective cation channel. The PC1-PC2 complex on the surface of cilia have been proposed to be activated by waves of fluid flow, which then transmits intracellular signals leading to mTORC1 activation[56] and calcium release from intracellular stores. Mutations in *PKD*1 or *PKD2* have been hypothesized to alter intracellular signaling and cell matrix interactions, resulting in kidney epithelial cell reorganization into spherical rather than tubular structures, which then fill with fluid and expand the developing cysts (see [57] for review). One model for how tubular epithelial cells reorganize from tubular to spherical morphology posits that loss of cell polarity (through changes in cell adhesion) causes epithelial cells to stop dividing along the axis parallel to the tubule lumen, and divide excessively in a circular pattern[57] (S3 Fig).

Increased division of renal tubular epithelial cells is driven in part by increased activation of mTORC1 (see [55, 58] for review), a major driver of cellular growth. Previous studies have shown a link between PC1 and mTORC1, whereby the cytoplasmic tail of PC1 interacts with Tuberin, the protein product of the *TSC2* gene. It has been proposed that PC1 sequesters the mTORC1 complex together with Tuberin, which in turn inhibits mTOR activity [59, 60]. Mutations in PC1 release Tuberin from mTOR, allowing mTORC1 to translocate to the lysosome where it is activated [61]. Another proposed mechanism for mTORC1 activation in ADPKD is due to co-deletion of the *TSC2* gene along with *PKD1*, since both are located adjacent to each other on the human chromosome 16[58]. In various murine models of ADPKD, mTORC1 is significantly hyper-activated in kidney tissue, and inhibition of mTORC1 with rapamycin can significantly inhibit or reduce the severity of PKD[59, 62, 63]. mTORC1 has also been shown to negatively regulate the production of PC-1 and trafficking of the PC-1/2 complex to the cilia[60]. In this study, we found that loss of *Fnip1* was sufficient to increase mTORC1 activation primarily in renal epithelial cells lining renal cysts. Increased mTORC1 activation in this context was associated with increased expression of cell adhesion molecules, which are known to effect cell polarity during division[57]. These results collectively suggest that loss of Fnip1 may contribute to PKD in part by increasing mTORC1 activation and upregulating expression of cell adhesion molecules, which together could alter cell polarity and promote cell division into spherical rather than tubular structures (S3 Fig).

Another major driver of increased epithelial cell division in ADPKD is the proto-oncogene c-Myc, whose expression is also increased in mouse models of PKD[55]. One study showed that transgenic expression of c-Myc is sufficient to cause cyst formation in primary murine kidneys[51]. Both c-Myc and mTOR are major drivers of the Warburg Effect, whereby cancer cells switch their metabolic preferences from oxidative phosphorylation, which produces mostly ATP, to aerobic glycolysis, which generates precursors such as amino acids, nucleic acids, and lipids required to fuel cell division (see [64] [49] for review). Importantly, increased aerobic glycolysis has been also linked to ADPKD [56, 65, 66]. Increased glycolysis in PKD is also through loss of LKB1, which functions in part to activate AMPK[67] and inhibit mTOR[56]. Kidney specific deletion of *Lkb1* results in impaired AMPK activation, excessive mTORC1 activation, and the development of renal cysts[67]. Treatment of kidney specific *Lkb1*-null mice with non-metabolizable 2-deoxyglucose (2-DG) inhibits glycolysis and limits cyst formation. Metformin, a pharmacological activator of AMPK, inhibits both CFTR and mTOR pathways, and significantly arrests the growth of renal cysts *in vitro* and *in vivo*[68]. In this study, we found that loss of *Fnip1* resulted in decreased AMPK activation; increased mTORC1 activation; increased oxidative phosphorylation, and increased expression of genes regulated by the mTORC1 targets PGC1alpha and Myc in *Fnip1*^*-/-*^ versus WT kidney tissue. The PGCalpha transcriptional regulator is a major driver of mitochondrial biogenesis and oxidative phosphorylation, whereas c-Myc stimulates glycolysis. These results are consistent with Fnip1 regulating epithelial cell proliferation and metabolism, in part through regulation of AMPK, PGCalpha, mTORC1, and c-Myc, which in turn stimulate mitochondrial biogenesis, OXPHOS, aerobic glycolysis, and cellular growth.

Other signaling pathways activated by the PC1/PC2 complex and are believed to modulate ADPKD, include activation of the Janus kinases (JAK) and Signal Transducers of Activated T cells (STAT/STAT3) transcription factors, which have been shown to both inhibit and stimulate renal epithelial cell division[69, 70]; activation of Ras/Raf/Erk signaling[71–73], which modifies proliferative and apoptotic stimuli; and activation of non-canonical Wnt signaling [74–76], which promotes cell polarity (for review see [57]). In this study, we found that disruption of *Fnip1* synergizes with *Tsc1* loss to increase Erk phosphorylation in *Fnip1*^*-/-*^*Tsc1*^*-/-*^ kidney tissue versus *Fnip1*^*-/-*^ or *Tsc1*^*-/-*^ kidney tissue, suggesting that the Ras/Raf/Erk pathway is activated. In addition, we found both Stat1 and Stat3 target genes were significantly decreased in *Fnip1* null versus WT kidney tissue. mRNA expression of Frizzled B (*Fzlb*), the receptor for Wnt, was significantly decreased and Wnt inhibitor factor 1 (*Wif1*) was significantly increased in *Fnip1* null versus WT kidneys. These results suggest that decreased Stat1/3 (which would result in decreased p21 and increased cell cycle progression[70]), decreased Wnt signaling (which would result in decreased polarity), and increased Erk signaling (which would promote mTORC1 activation by inhibitory phosphorylation of TSC2[77]) may contribute to the development of PKD following disruption of *Fnip1*.

Following reorganization of epithelial cells into spherical cysts due in part to excessive cell proliferation, conversion from an ion absorptive to an ion secretory epithelium has been proposed to cause ion secretion into the cyst lumen, resulting in passive fluid accumulation[57, 78] (S3 Fig). In ADPKD, the ion secretory epithelium is defined by many changes in expression of ion channels including the cAMP activated cystic fibrosis transmembrane receptor (CFTR) chloride channel, the Na^+^-K^+^-2Cl^-^ (NKCCl) co-transporter encoded by *slc12a1* and -*a2*, and Ca^2+^ cation channels, among others[79–81]. AMPK also negatively regulates the CFTR and Cl^-^ secretion, and activation of AMPK with metformin inhibits CFTR secretion and reduces cyst growth[82–85]. Although the roles of other ion transporters in ADPKD have not been specifically addressed, alterations in the expression of any ion transporter that results in changes in ion concentrations in the cyst lumen could alter cyst development. In this study, we found that loss of *Fnip1* significantly alters the expression of numerous genes encoding cation and anion transporters; of the 206 genes down-regulated following disruption of *Fnip1*, we found the highest enrichment for genes associated with sodium-independent ion transport, organic ion transport, and anion transmembrane transport. These results suggest that one of the functions of Fnip1 is to control ion transport, perhaps through AMPK activation, which could play a role in the development of PKD following disruption of *Fnip1*.

More recently, the roles of inflammatory cell infiltration in the development of ADPKD have been investigated with compelling results. In particular, interstitial inflammation characteristic of ADPKD and has been shown to result in elevated levels of cytokines and chemokines in the renal cyst fluid of patients with ADPKD, which is thought to contribute to disease progression[86–88]. Alternatively activated “M2-like” anti-inflammatory kidney macrophages, which are uniquely situated in close apposition to endothelial cells and basement membrane, have been shown to promote cyst growth in ADPKD by driving proliferation and fibrosis in the cystic epithelium, in part by induction of complement components including C3, TGF-ß, and connective tissue growth factor (CTGF)[54, 89, 90]. Classically activated “M1” pro-inflammatory macrophages also contribute to epithelial cell damage by producing ROS and pro-inflammatory cytokines. Importantly, depletion of macrophages was shown to inhibit epithelial cell proliferation and cyst growth and improve renal function[54, 91]. In addition, PKD susceptible mice switched into a germfree environment fail to form cystic kidneys[92]. These results suggest that immune cell infiltration can play a unique and important role in the pathogenesis of PKD by producing pro-inflammatory mediators in response to tissue damage. Our results suggest that disruption of *Fnip1* alone results in increased kidney inflammation, specifically in the regions surrounding cysts. By IHC, we see enrichment of F4/80^+^ macrophages immediately surrounding cysts, and CD3^+^ T cells in similar regions. Using RNAseq and quantitative PCR, we found significant enrichment for genes involved in immune responses, including genes encoding complement components, Toll-like and NOD-like receptors, Fc-receptors, and S100 pro-inflammatory proteins. By flow cytometry, we found significant enrichment in the representation of myeloid cells in *Fnip1*^*-/-*^ kidneys. These results suggest that Fnip1 may normally suppress immune cell infiltration into the kidney parenchyma, which may function to limit inflammation and renal epithelial cell activation.

We also found that disruption of *Fnip1* potently synergizes with *Tsc1* loss to hyperactivate mTORC1 in otherwise normal renal tissue. This result further fuels the confusion as to whether the Fnip1/Fnip2/Folliculin complex is important for activating or inhibiting mTOR. Using siRNA knockdown approaches in human cell lines, two groups have presented evidence that Folliculin/Fnip recruits mTOR to the lysosome, where it is activated in response to amino acid stimulation [24, 25]. The conclusion made from these studies was that the Flcn/Fnip complex is necessary for GTP/GDP exchange in order to activate the Rag GTPases and to permit mTOR recruitment to the lysosome for activation. However, our data shows that loss of Fnip1 in kidney tissue results in increased activation of mTOR, and that loss of *Fnip1* synergizes with loss of *Tsc1* to further hyperactivate mTOR and greatly accelerate the development of PKD. Hasumi et al. found that kidney specific disruption of both *Fnip1* and *Fnip2* together using cadherin16-Cre (*CDH16*-Cre) resulted in severe PKD (50% survival around 21 days) characterized by increased activation of mTORC1and PGC1alpha. These results indicate that there is considerable functional compensation for the absence of Fnip1 by Fnip2 (and visa versa) either because their molecular functions are identical, or because disruption of *Fnip1* and *Fnip2* potently synergize to cause PKD due to activation of unique but cooperating pathways. Interestingly, although cyst formation was not observed in single *Fnip1*^*fl/fl*^ *CDH16*-Cre or *Fnip2*^*fl/fl*^ *CDH16*-Cre kidneys, the authors did find increased kidney/body weight and small cyst formation in *Fnip1*^*fl/fl*^ *Fnip2*^*fl/+*^ *CDH16-Cre* kidneys (31). The differences between our study and Hasumi et al may reflect differences in genetic background, or the use of kidney specific disruption of *Fnip1* by *Hasumi et al* versus constitutive disruption of *Fnip1* in our study. These results collectively suggest that Fnip1 may normally function in renal epithelial cells to limit activation of mTOR in this context. One potential model for how Fnip1 loss and Tsc1 loss may synergize to hyperactivate mTORC1, is that loss of Fnip1/Fnip2/Flcn may result in increased recruitment of mTOR to the lysosome where it can be activated by Rheb, which is negatively regulated by TSC1. Consistent with this model, we also find that *Fnip1*^*-/-*^ skeletal muscle tissue[12] and pre-B cells[18] also exhibit hyperactivation of mTOR, and another laboratory has shown that AMPK via interaction with Axin and LKB1, is tethered with Fnip1/Flcn at the lysosomal surface in response to low glucose, where it displaces mTORC1[93]. Loss of Fnip1/Flcn is predicted to inhibit AMPK recruitment and activation at the lysosome and facilitate mTORC1 recruitment and activation. Indeed, we find decreased AMPK activation and increased mTORC1 activation in *Fnip1* null renal tissue.

A major question is how Fnip1 controls so many molecular programs that, when dysregulated, may contribute to the development of PKD. One potential model is that Fnip1 controls the differentiation state of renal epithelial cells[94]. For example, it has been shown that renal gene expression differs considerably before and after postnatal day 13, when cell gene expression shifts abruptly from predominately proliferative to expression of genes also involved in cell polarity, extracellular matrix composition, and inflammation[95]. Perhaps loss of Fnip1 inhibits renal epithelial cells from adopting a more mature cell fate, thus altering their response to injury or mTOR activation, thus promoting cystogenesis. Consistent with this model, loss of Fnip1 inhibits differentiation of B lymphocytes[18, 96], invariant NK T cells[97], and embryonic stem cells[98], indicating that Fnip1 is linked to cellular differentiation in some cellular contexts.

## Supporting information

S1 Fig Disruption of Fnip1 results in increased mTORC1 signaling and decreased AMPK signaling in kidney cortical tissue. (A) Shown is quantitation of immunoblots in Fig 2A using densitometry followed by ImageJ analyses. * = p<0.05 (B) Immunoblots showing expression of Fnip1, Fnip2, and Folliculin protein in *Fnip1*^*-/-*^ and WT mice. Each lane represents 3 individual mice of the indicated genotype.
(TIFF)

S2 Fig Electron microscopy on kidney tissue from *Fnip1* null and wildype mice. Kidney tissue from 3 wildtype and 3 *Fnip1*^*-/-*^ mice were submitted for transmission EM. (A) Shown is a representative image of renal tubular epithelial cells. (B) Percent mitochondrial area is shown. Differences were not statistically significant.
(TIFF)

S3 Fig Model of Fnip1 functions in kidney. **(A)** Proposed regulation of AMPK and mTOR by Fnip1. Fnip1 regulates the abilities of LKB1 and/or CamKK to activate AMPK. Fnip1 may regulate the localization activation of mTOR at the lysosome. **(B)** Model for Fnip1 functions in renal tissue. A number of cellular and molecular events have been linked to increased propensity to develop PKD. Shown of a proposed model for how loss of *Fnip1* could lead to the development of renal cysts.
(TIFF)

S4 Fig Disruption of Fnip1 results in increased mTORC1 and increased Erk signaling in kidney cortical tissue. Shown is quantitation of immunoblots in Figs 7E and 8D using densitometry followed by ImageJ analyses. Significant P-values are shown.
(TIFF)

S1 Table Metabolites significantly different between *Fnip1* null and wildtype kidney tissue as measured by liquid chromatography mass spectrometry (LC-MS/MS). Data were analyzed using Metaboanalyst 3.0.
(TIFF)

S2 Table Selected genes differentially expressed between *Fnip1* null and wildtype kidney tissue, as determined by RNAseq analyses. (TIFF)

S3 Table Ingenuity Upstream Regulator analyses showing disruption of *Fnip1* results in increased representation of specific transcriptional target genes. (TIFF)

S4 Table Oligonucleotide sequences used for quantitative PCR. (TIFF)
