# Supplementary figures and images for: Loss of Fnip1 alters kidney developmental transcriptional program and synergizes with TSC1 loss to promote mTORC1 activation and renal cyst formation
Source: PLoS One. 2018 Jun 13;13(6):e0197973. doi: 10.1371/journal.pone.0197973 (PMC5999084; doi:10.1371/journal.pone.0197973)

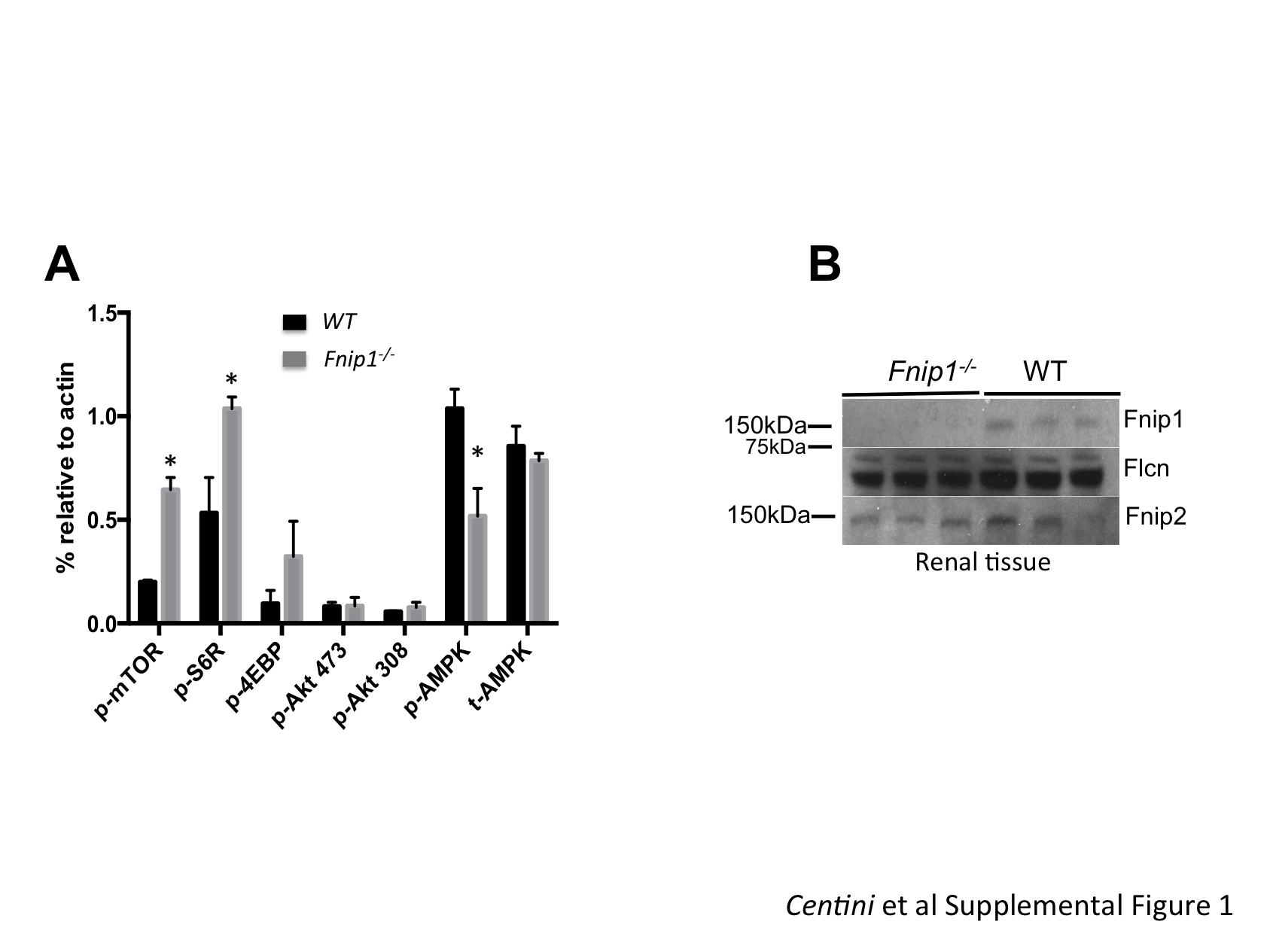

Supplement: S1 Fig — (A) Shown is quantitation of immunoblots in Fig 2A using densitometry followed by ImageJ analyses. * = p<0.05 (B) Immunoblots showing expression of Fnip1, Fnip2, and Folliculin protein in Fnip1-/- and WT mice. Each lane represents 3 individual mice of the indicated genotype. (TIFF) [file pone.0197973.s001.tiff]

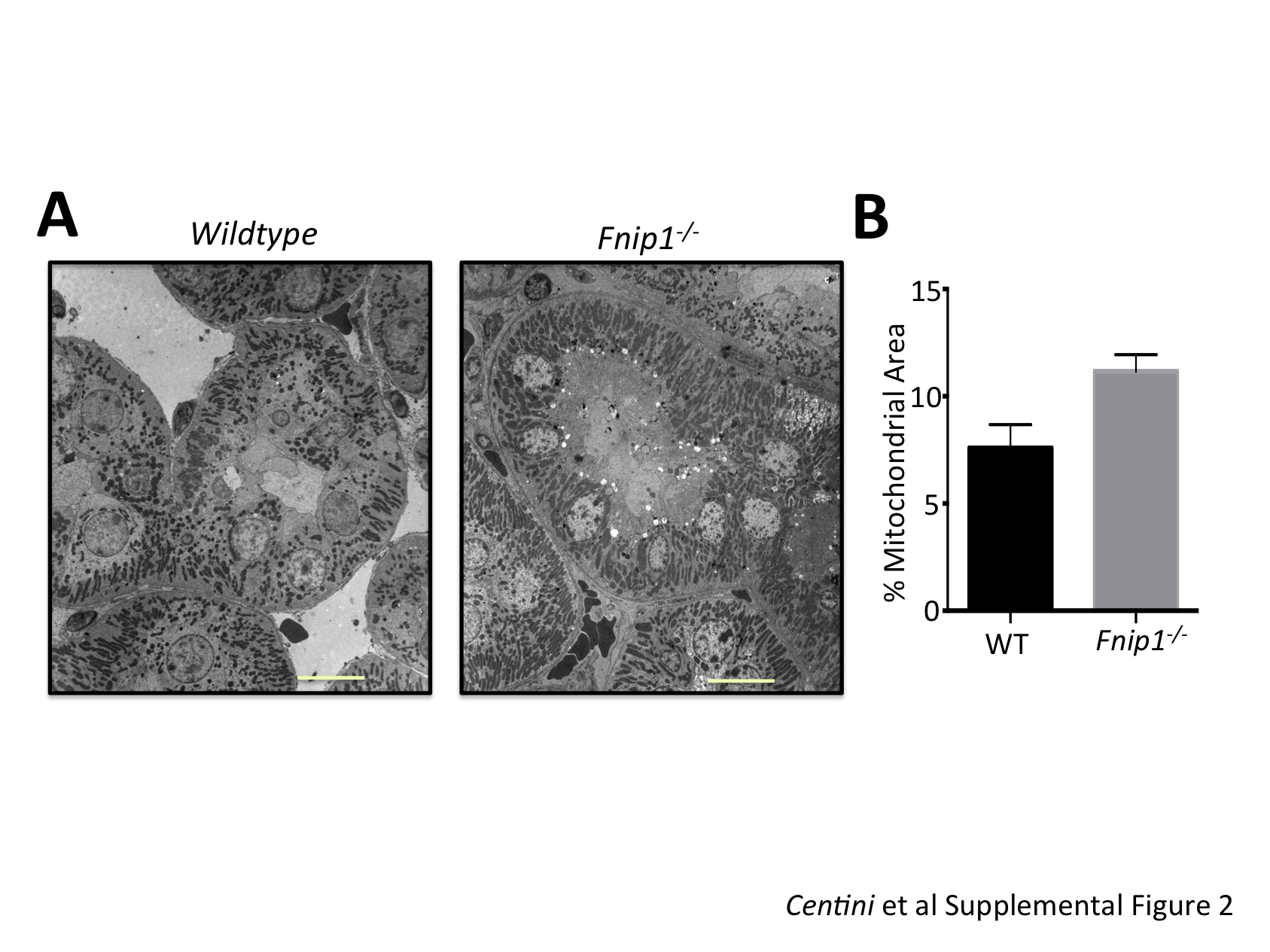

Supplement: S2 Fig — Kidney tissue from 3 wildtype and 3 Fnip1-/- mice were submitted for transmission EM. (A) Shown is a representative image of renal tubular epithelial cells. (B) Percent mitochondrial area is shown. Differences were not statistically significant. (TIFF) [file pone.0197973.s002.tiff]

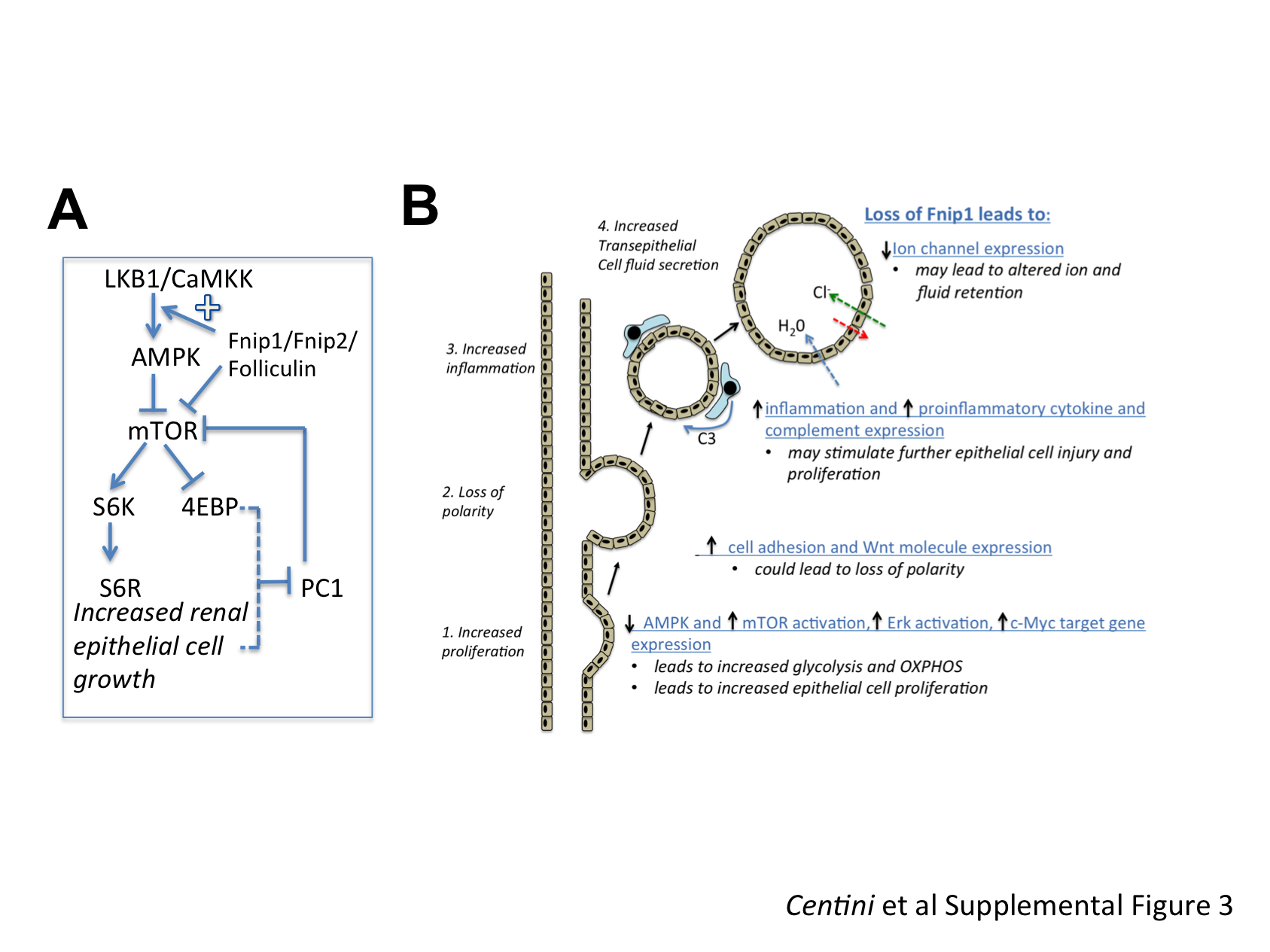

Supplement: S3 Fig — (A) Proposed regulation of AMPK and mTOR by Fnip1. Fnip1 regulates the abilities of LKB1 and/or CamKK to activate AMPK. Fnip1 may regulate the localization activation of mTOR at the lysosome. (B) Model for Fnip1 functions in renal tissue. A number of cellular and molecular events have been linked to increased propensity to develop PKD. Shown of a proposed model for how loss of Fnip1 could lead to the development of renal cysts. (TIFF) [file pone.0197973.s003.tiff]

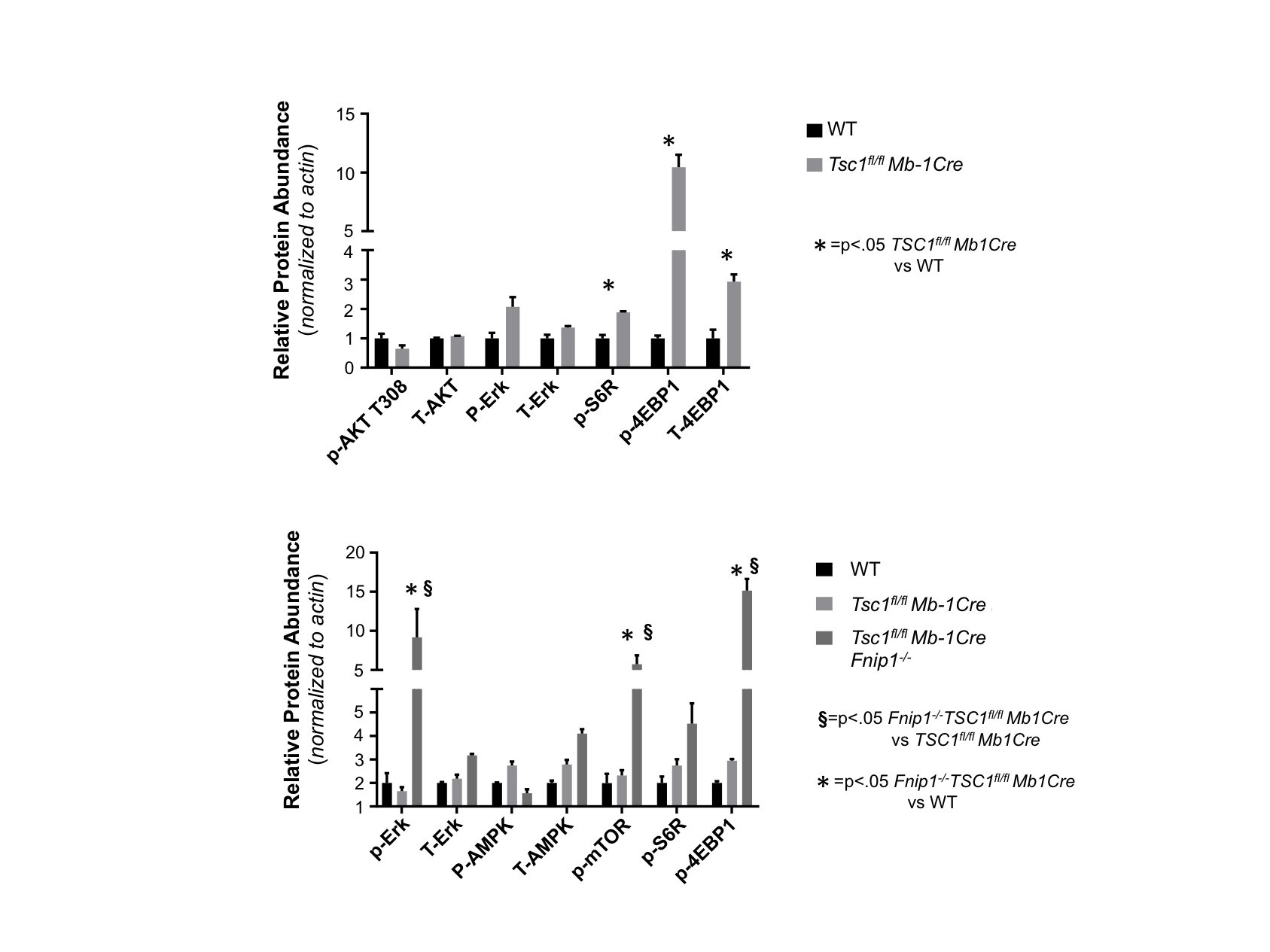

Supplement: S4 Fig — Shown is quantitation of immunoblots in Figs 7E and 8D using densitometry followed by ImageJ analyses. Significant P-values are shown. (TIFF) [file pone.0197973.s004.tiff]

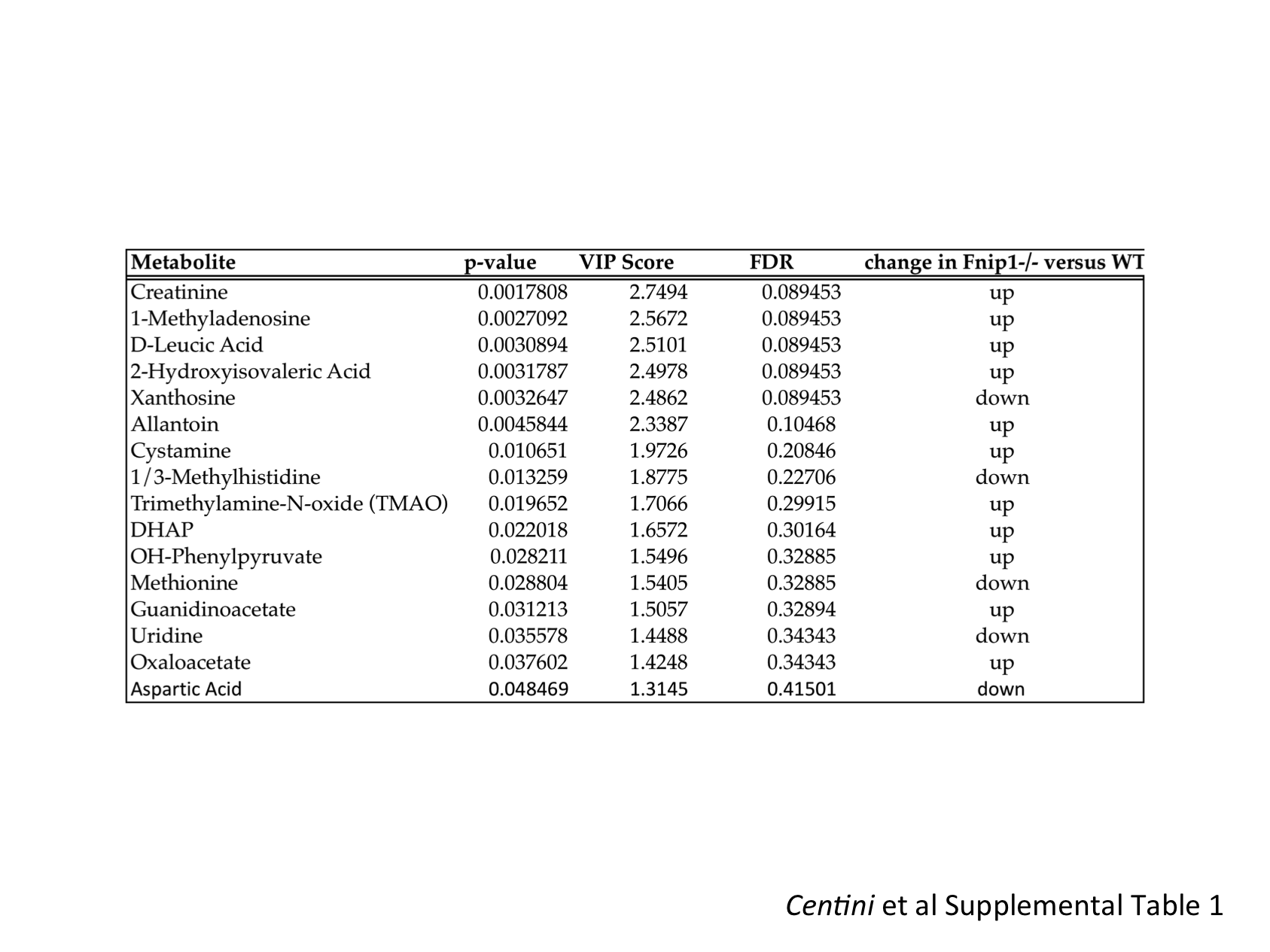

Supplement: S1 Table — Data were analyzed using Metaboanalyst 3.0. (TIFF) [file pone.0197973.s005.tiff]

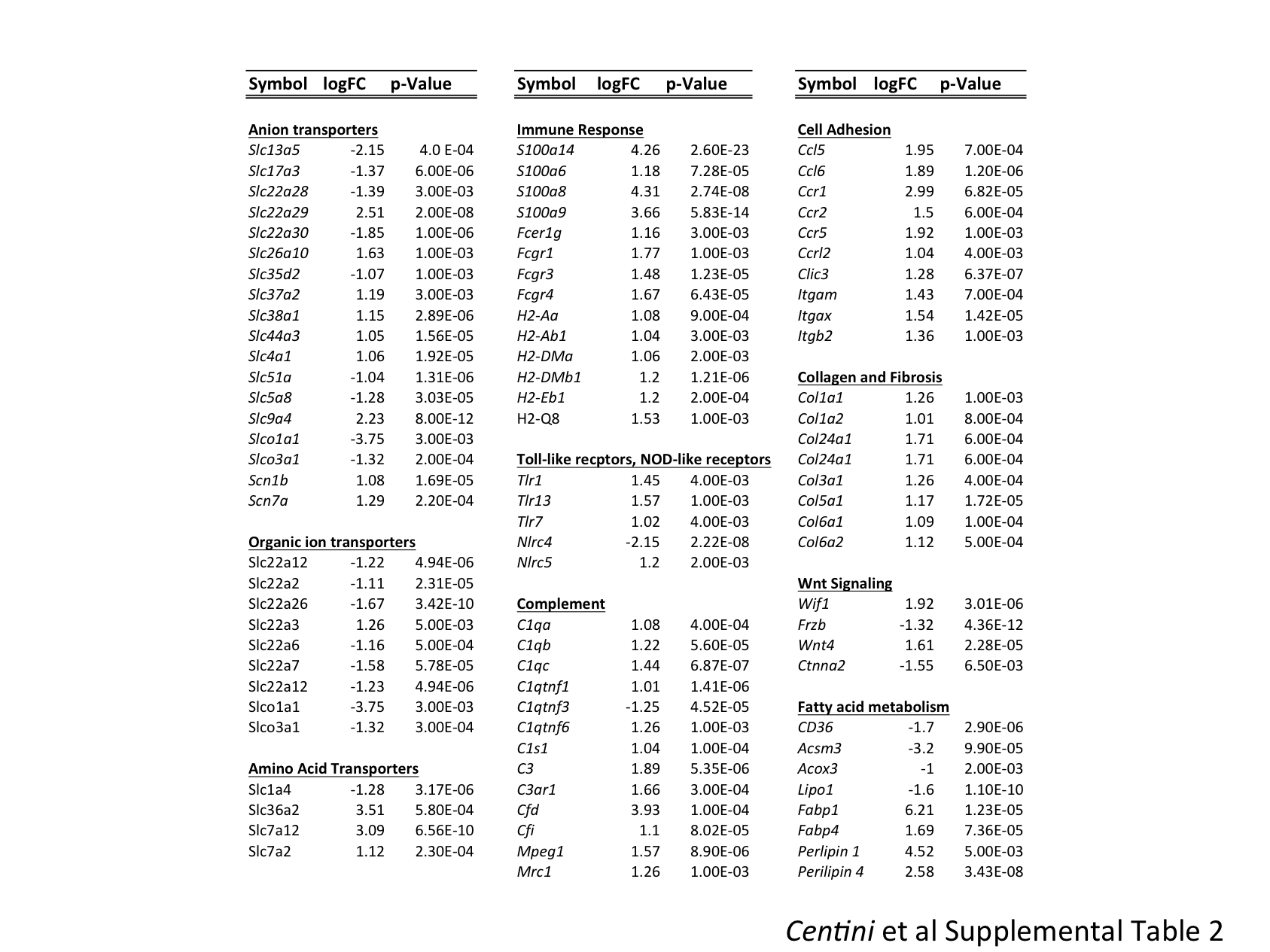

Supplement: S2 Table — (TIFF) [file pone.0197973.s006.tiff]

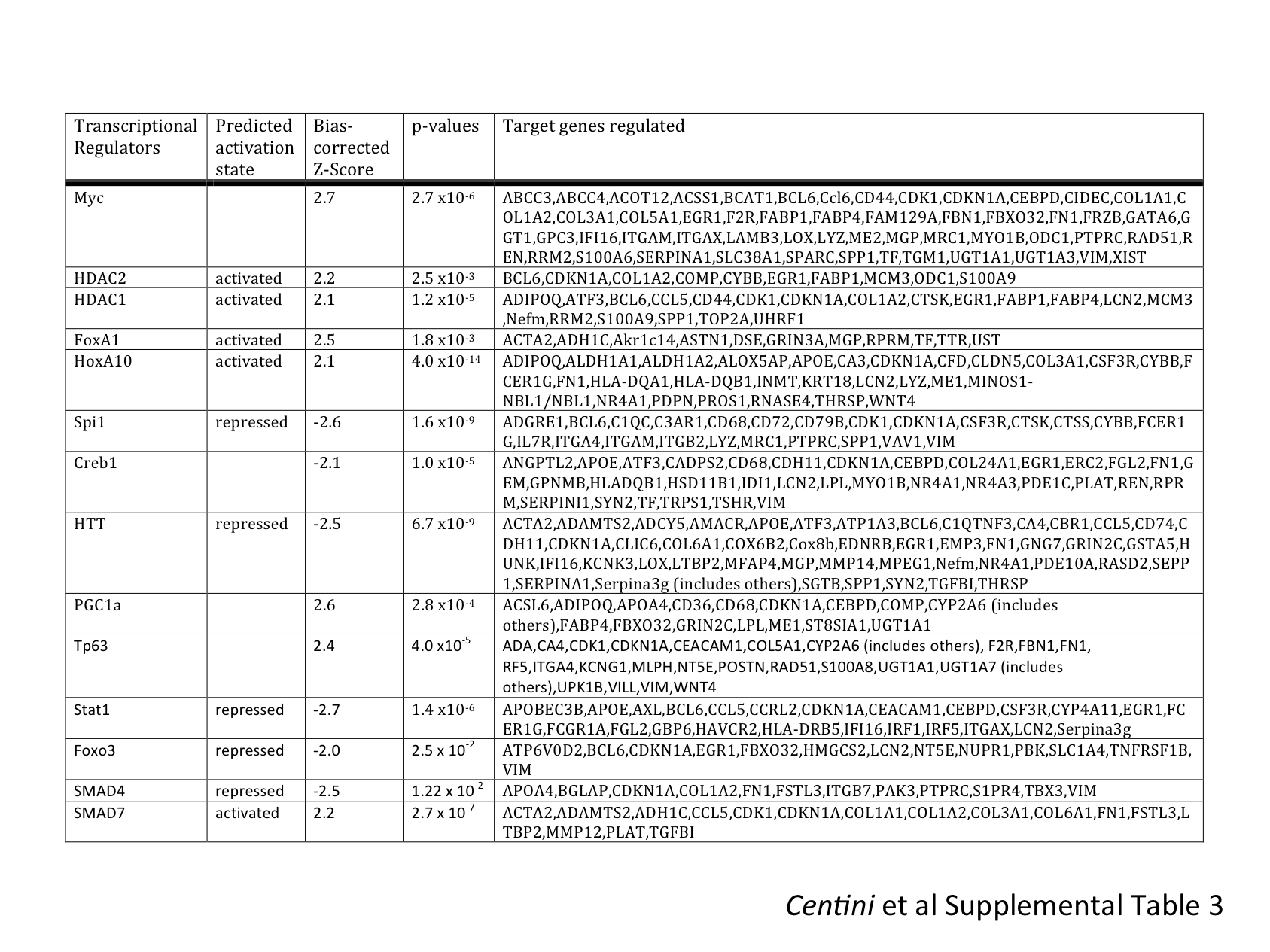

Supplement: S3 Table — (TIFF) [file pone.0197973.s007.tiff]

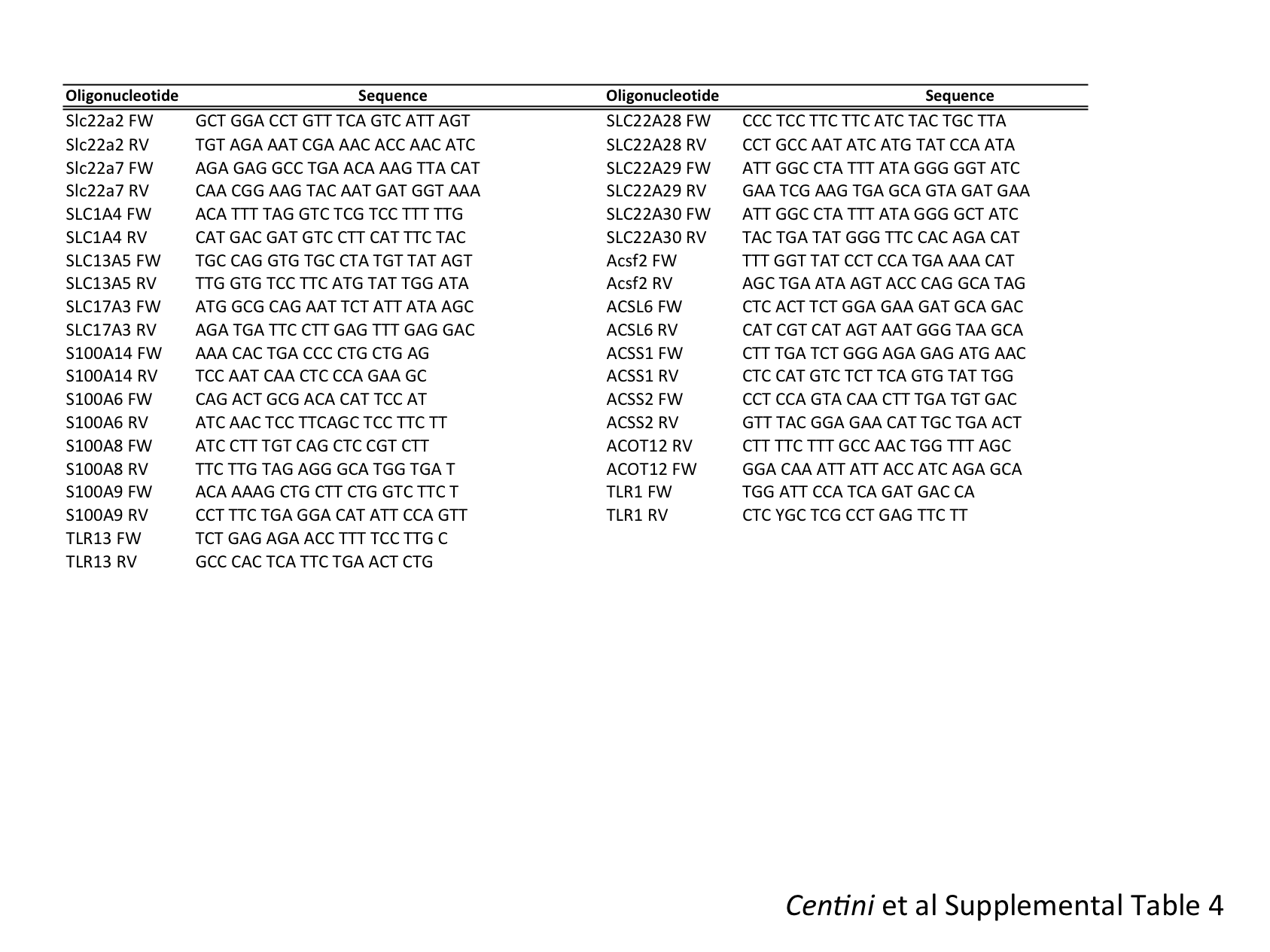

Supplement: S4 Table — (TIFF) [file pone.0197973.s008.tiff]
